# Nagilactone C from the Seeds of *Podocarpus nakaii* May Protect Against LPS-Induced Acute Lung Injury via STAT Signaling Pathway Inhibition

**DOI:** 10.3390/ph18091319

**Published:** 2025-09-03

**Authors:** Xiaoxiao Chen, Jing Tang, Shijie Zhan, Yixian Qiu, Jing Li, Weiguang Shan, Youmin Ying

**Affiliations:** 1 College of Pharmaceutical Science, Zhejiang University of Technology, Hangzhou 310014, China; angel_xiao85@163.com (X.C.); t_joyce@126.com (J.T.); zhanshijie@zjut.edu.cn (S.Z.); 13588779931@163.com (Y.Q.); 221123070147@zjut.edu.cn (J.L.); swg@zjut.edu.cn (W.S.); 2 Pharmaceutical Engineering College, Jinhua University of Vocational Technology, Jinhua 321017, China

**Keywords:** acute lung injury, *Podocarpus nakaii*, nagilactone C, anti-inflammatory, STAT

## Abstract

**Background/Objectives:** Acute lung injury (ALI) is a respiratory disorder lacking specific targeted therapy. Our preliminary screening revealed that the ethanol extract of the seeds of *Podocarpus nakaii* (EESPN) alleviated the symptoms of ALI in mice. The objectives of this study were to identify the active constituents in EESPN and study the mechanism involved. **Methods:** Column chromatography was performed to separate the chemical constituents of EESPN. The structures of the isolates were determined via spectroscopic methods. MTT assays, Western blotting, histological analysis, TUNEL assays, immunofluorescence staining, transcriptomic analysis, and quantitative real-time polymerase chain reaction (qRT–PCR) were employed to evaluate the anti-inflammatory activity and to elucidate the potential mechanism of nagilactone C (**3**, Nag C) in ALI treatment. **Results:** Twelve compounds were isolated from EESPN and structurally characterized. The structure of podolactone E (**1**) was confirmed via single-crystal X-ray diffraction. In vitro, Nag C showed potent anti-inflammatory activity in LPS-induced RAW 264.7 cells. Nag C liposomes significantly ameliorated LPS-induced histopathological damage to the lungs, reduced neutrophil infiltration and inflammatory cytokine levels, increased myeloperoxidase (MPO) activity, and promoted apoptosis in mice. In addition to suppressing inflammation, Nag C also significantly suppressed the phosphorylation of the NF-κB, STAT3, and STAT1 proteins. **Conclusions:** Nag C is an active constituent of EESPN. It may protect against LPS-induced ALI via inhibition of the STAT signaling pathway. Thus, Nag C is a promising lead compound in the development of novel STAT-targeted anti-inflammatory agents.

## 1. Introduction

Acute lung injury (ALI) and its severe form, acute respiratory distress syndrome (ARDS), represent critical respiratory emergencies frequently encountered in clinical practice and are characterized by diffuse alveolar damage, pulmonary edema, and refractory hypoxemia [1,2,3]. Acute respiratory distress syndrome (ARDS) is characterized by noncardiogenic pulmonary edema. It has a high mortality rate and lacks effective pharmacotherapy [4]. In COVID-19, cytokine release syndrome can cause severe lung tissue damage leading to acute respiratory distress syndrome (ARDS) [5]. With the outbreak of COVID-19 worldwide, the mortality of ARDS has increased correspondingly, which makes it urgent to find effective targets and strategies for the treatment of ARDS. Recent clinical trials of Janus kinase (JAK) inhibitors in treating COVID-19-induced ARDS have shown a positive outcome, which makes the Janus kinase/signal transducer and activator of transcription (JAK/STAT) pathway a potential therapeutic target for treating ARDS [4].

ALI has a rapid onset and represents an early stage of ARDS. Without timely and effective treatment, ALI may progress to ARDS, resulting in irreversible lung injury and, potentially, death [2]. Despite substantial advances in ventilatory support techniques and intensive care management in recent years, ALI/ARDS mortality remains alarmingly high at approximately 40%, imposing a significant epidemiological burden on global public health systems [6,7,8]. ALI is pathologically characterized by the infiltration of inflammatory cells and an increase in pro-inflammatory mediators, leading to disruption of the alveolar–capillary barrier and pulmonary edema, which ultimately results in severe impairment of gas exchange [9,10]. Hence, anti-inflammatory interventions are critical for preventing ALI progression to ARDS. Although aberrant pulmonary inflammation underlies the pathophysiology of ALI, there are currently no targeted pharmacotherapies available. Clinical management relies on supportive care such as mechanical ventilation combined with corticosteroids. This approach has limited efficacy and significant adverse effects [11,12]. Therefore, there is an urgent need to identify novel, highly effective anti-inflammatory agents with reduced adverse effects.

*Podocarpus* is the most representative genus within the family *Podocarpaceae* and is phylogenetically related to the *Taxaceae* family. Traditional medicine systems in diverse regions, including China (notably Yao medicine), Africa, and New Zealand, utilize plants of *Podocarpus* species, such as *P. nagi*, *P. sensu latissimo* (s. l.), and *P. totara*, for their therapeutic properties [13]. Phytochemical investigations in recent decades have revealed that terpenoids, flavonoids, steroids, and phenols constitute the predominant classes of compounds isolated from various *Podocarpus* species, with terpenoids being the most abundant [13,14,15]. Notably, norditerpene dilactones, a subclass of terpenoids, have received the greatest amount of pharmacological research attention [15]. These phytochemicals were reported to exhibit diverse bioactivities, including anti-inflammatory, anticancer, antimicrobial, and antioxidant activities, with the anti-inflammatory activity being particularly notable [13,14,15,16,17,18,19]. Evidence from cytokine-based inflammation models has indicated that *Podocarpus* extracts exhibited significant anti-inflammatory activity, mediated by their inhibitory effects on various signal transduction pathways [13]. However, previous phytochemical investigations have been limited in number and have disproportionately focused on *P. nagi* and *P. macrophyllus*, with many congeners receiving minimal attention.

*P. nakaii* is present sporadically in broadleaved (angiosperm) forests in the central mountains of Taiwan [20]. It was rated as endangered according to the IUCN Red List criteria. Hence, phytochemical studies on this species are limited. Our preliminary screening revealed that the ethanol extract of the seeds of *P*. *nakaii* (EESPN) alleviated the symptoms of ALI in mice. In this study, EESPN was subjected to extensive chemical investigation, which led to the isolation and characterization of twelve compounds **1**–**12**. In the in vitro bioassay, nagilactone C (**3**, Nag C) showed potent anti-inflammatory activity, which was further systematically investigated for in vivo efficacy and mechanisms of action. Simultaneously, a three-stage research strategy—”transcriptome-wide profiling → targeted pathway validation → causal cellular verification”—was implemented to identify and validate molecular targets.

## 2. Results

### 2.1. Separation and Structural Characterization of Compounds Derived from EESPN

**Figure 1:**
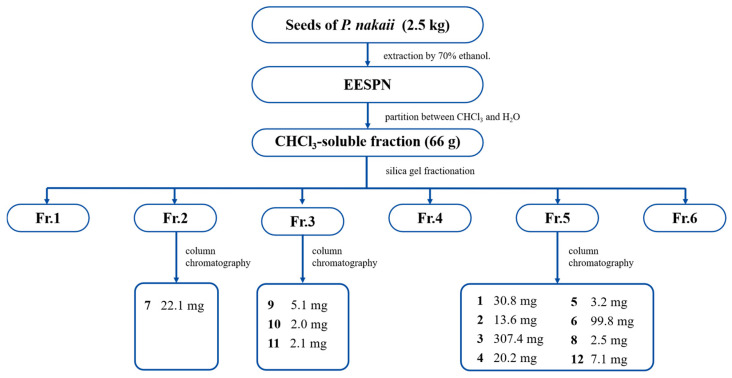
Flowchart for the separation of compounds **1**–**12**.

EESPN was partitioned between chloroform and water to yield a chloroform-soluble fraction, which was separated by repeated column chromatography to yield twelve compounds **1**–**12** (Figure 1).

**Figure 2:**
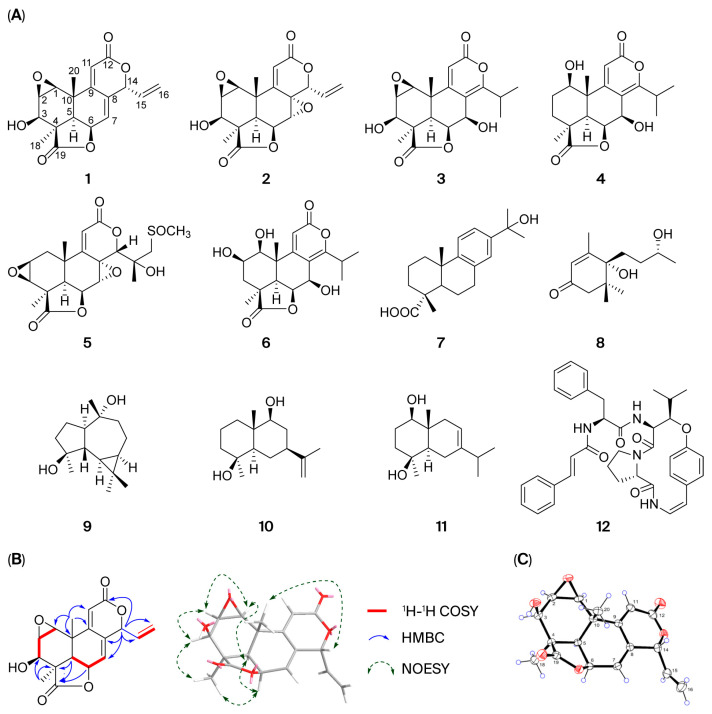
Separation and identification of the chemical constituents of EESPN. ((**A**). Chemical structures of compounds **1**–**12**. (**B**). Key ^1^H-^1^H COSY, HMBC, and NOESY correlations for **1**. (**C**). ORTEP drawing of **1**).

Compounds **1**–**12** were identified as podolactone E (**1**), inumakilactone B (**2**) [21], Nag C (**3**) [22,23], nagilactone A (**4**) [22,24], podolactone C (**5**) [25,26], nagilactone B (**6**) [27], 15-hydroxydehydroabietic acid (**7**) [28], dihydrovomifoliol (**8**) [29], 4*β*,10*α*-aromadendranediol (**9**) [30], eudesma-11(3)-ene-4*β*,9*β*-diol (**10**) [31], oplodiol (**11**) [32], and neriitide A (**12**) [33] by comparison of the spectroscopic data with those reported in the literature (Figure 2A). Notably, a SciFinder database search revealed that podolactone E corresponded to two diastereoisomers sharing a common set of NMR data. One isomer, reported by Galbraith et al. in 1972 from *P. neriifolius* D. Don ex Lamb., is characterized by an *α*-oriented 1,2-epoxy group [34]. The other isomer, documented in references [23,35], possessed a *β*-oriented 1,2-epoxy group. This prompted us to study the structure of **1** by extensive spectroscopic methods. Comprehensive elucidation of the ^1^H-^1^H COSY, HSQC, and HMBC spectra (Figure 2B) revealed that **1** possessed the same planar structure as podolactone E [23,34,35], bearing a podolactone skeleton. The relative stereochemistry of **1** was determined by NOESY experiments. For podolactones, H-5 is generally defined as being in the *α*-orientation. In the NOESY spectrum of **1**, both H-1 (*δ*_H_ 3.54) and H-2 (*δ*_H_ 3.49) showed apparent correlations with H-5 (*δ*_H_ 2.14), suggesting that the 1,2-epoxy group in **1** is *β*-oriented. Fortunately, we obtained suitable single crystals of **1** in methanol. Single-crystal X-ray diffraction (**Cu-Kα**) analysis (CDCC deposit number: 2130852) verified the relative stereochemistry and established the absolute configurations as 1*S*, 2*R*, 3*R*, 4*R*, 5*R*, 6*S*, 10*S*, 14*R* (Figure 2C).

### 2.2. In Vitro Anti-Inflammatory Assay

In this study, all the isolated compounds were evaluated for their in vitro anti-inflammatory activity in RAW 264.7 mouse macrophages. Cell viability was first assessed by treating the cells with compounds (0.097–50 μM) for 24 h via MTT assay. The results revealed that the half-maximal inhibitory concentration (IC_50_) of compounds **1**–**12** toward RAW264.7 cells were 0.3911, 0.2849, 5.951, 11.68, 28.64, 31.62, >50, >50, >50, >50, >50, and 49.3 μM, and the maximum nontoxic doses of compounds **1**–**12** were 0.097, 0.097, 0.391, 3.125, 6.25, 3.125, >50, 3.125, 6.25, 3.125, 6.25, and 1.563 μM, respectively (Figures S31–S33).

To investigate the therapeutic effects of compounds **1**–**12** on lipopolysaccharide (LPS)-induced inflammation in RAW264.7 cells in vitro, we first set three non-toxic doses for each compound on the basis of the cytotoxicity results. The effects of the 12 compounds on the levels of IL-6, IL-1β, and TNF-α in the cells were subsequently assessed via RT–qPCR to evaluate their anti-inflammatory activities. LPS induction significantly increased the levels of IL-6, IL-1β, and TNF-α in RAW264.7 cells compared with those in the control group (*p* < 0.01). Furthermore, all three inflammatory markers were significantly lower in the positive control group than in the model control group (*p* < 0.01), indicating that the experimental system was stable and reliable. Following drug treatment, only Nag C significantly decreased the mRNA levels of IL-6, IL-1β, and TNF-α (*p* < 0.01 vs. those in the model control group) in a dose-dependent manner, indicating the anti-inflammatory potential of Nag C. Therefore, the anti-inflammatory mechanisms of Nag C were assessed in subsequent experiments.

### 2.3. Characterization of Nag C Liposomes

**Figure 3:**
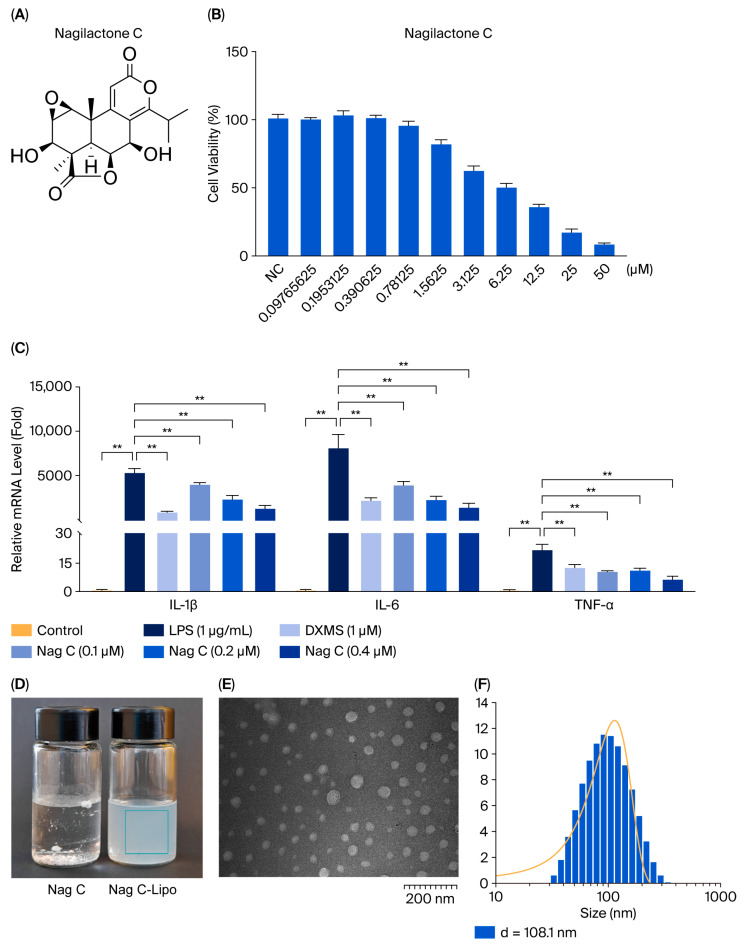
In vitro anti-inflammatory activity of Nag C and preparation of Nag C liposomes. ((**A**). Chemical structure of Nag C. (**B**). Cytotoxicity of Nag C against RAW264.7 cells (*n* = 3). (**C**). Effect of Nag C on the mRNA expression of IL-1β, IL-6, and TNF-α in LPS-induced RAW264.7 cells (*n* = 3). (**D**). Macroscopic morphology of the prepared Nag C liposome formulation (magnification: ×40,000; accelerating voltage: 90.0 kV). (**E**). Structural analysis of Nag C liposomes by TEM. (**F**). Dynamic light scattering (DLS) measurement of Nag C liposome hydrodynamic diameter (The orange line: frequency distribution curve of particle size). The above data are expressed as the means ± SDs. **, *p* < 0.01, vs. cells in the model group.).

The exceptional anti-inflammatory efficacy of Nag C (Figure 3A–C) prompted us to investigate its mechanistic basis. To overcome the dual challenges of drug scarcity (307.4 mg) and hydrophobicity, a sterically stabilized nanoliposome system was prepared for enhanced pulmonary delivery via the intranasal route to target ALI pathophysiology. Physicochemical characterization of the Nag C liposomes revealed a monodisperse spherical morphology via transmission electron microscopy (TEM). Quantitative analysis revealed favorable pharmaceutical parameters: average diameter (108 ± 15 nm, *n* = 50), zeta potential (−15 to −24 mV), and encapsulation efficiency (85.6%) (Figure 3D–F). The prepared Nag C liposomes exhibited no phase separation compared to their initial state following one week of storage at 4 °C, demonstrating satisfactory encapsulation efficiency.

### 2.4. Nag C Liposomes Significantly Ameliorated LPS-Induced ALI in Mice

**Figure 4:**
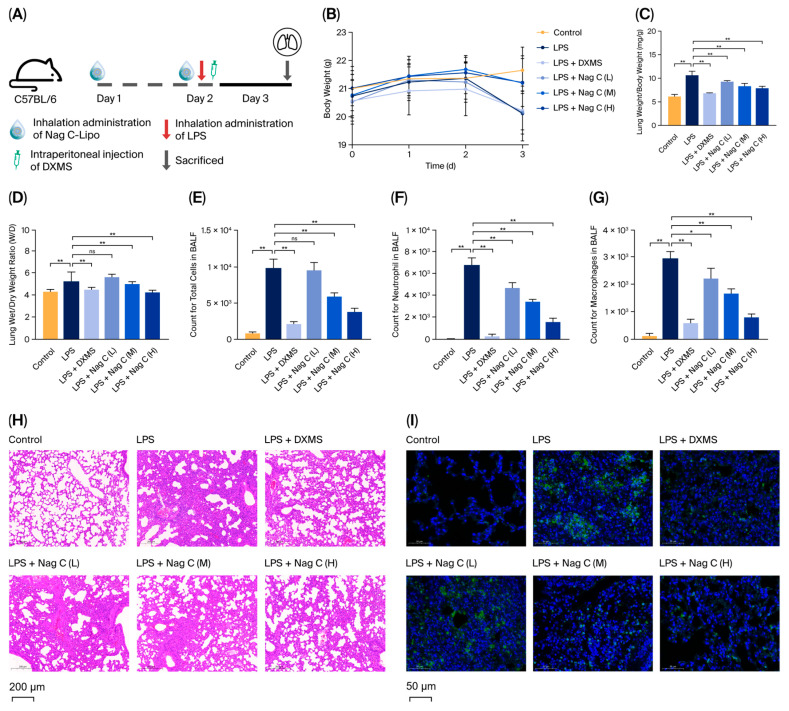
Protective effect of Nag C liposomes against LPS-induced ALI. ((**A**). Animal study protocol. (**B**). Body weight change curves (*n* = 8). (**C**). Lung indices of the mice (*n* = 8). (**D**). Lung wet–dry weight ratio (W/D ratio) (*n* = 3). (**E**). Total cell count in the BALF (*n* = 3). (**F**). Neutrophils in the BALF (*n* = 3). (**G**). Macrophage count in the BALF (*n* = 3). (**H**). Lung tissue histopathology (HE), scale bar: 200 μm. (**I**). Immunofluorescence staining for MPO expression levels in the mouse lung tissue, scale bar: 50 μm. The above data are expressed as the means ± SDs. *, *p* < 0.05, **, *p* < 0.01, ns = not significant vs. cells in the model group).

To investigate the in vivo therapeutic effects of Nag C liposomes on ALI, mice were pretreated with intranasal inhalation of the formulation 24 h prior to LPS challenge. A booster dose was administered 4 h post-induction, followed by comprehensive inflammatory profiling at 24 h (Figure 4A). As shown in Figure 4B, significant weight loss was observed in the LPS-induced model group (20.2 g) compared with the control group (21.5 g, *p* < 0.05) at 24 h post-induction. Notably, Nag C liposome treatment attenuated this LPS-induced weight reduction, with the high-dose and medium-dose groups showing significantly greater body weights than the model control group by day 3 (*p* < 0.05). Furthermore, LPS challenge significantly increased the lung organ coefficient. Nag C liposome treatment effectively reversed this increase. Notably, the high-dose group exhibited the most pronounced therapeutic effect (*p* < 0.01).

The lung wet–dry weight ratio (W/D ratio) represents a quintessential biomarker for the quantitative assessment of pulmonary edema in murine models, providing an objective measurement of alveolar–capillary barrier integrity through precise gravimetric analysis of pulmonary fluid content. The results revealed a significant increase in the lung W/D ratio in the LPS-challenged group compared with the control group (*p* < 0.01), indicating the development of substantial pulmonary edema following 24 h of LPS exposure. Compared with LPS treatment, dexamethasone (DXMS) treatment significantly attenuated the LPS-induced increase in the W/D ratio (*p* < 0.01), demonstrating both the robustness of the experimental system and the therapeutic efficacy of the intervention (Figure 4C). Compared with that in the LPS group, the W/D ratio significantly decreased in both the high-dose and medium-dose Nag C liposome groups (*p* < 0.01), indicating a clear dose-dependent relationship (Figure 4D). During pulmonary infection, various immune cells participate in host defense responses through mechanisms including pathogen recognition, inflammatory mediator release, and immune regulation. Evaluation of pulmonary inflammation by bronchoalveolar lavage fluid (BALF) cell counting revealed that, compared with the control condition, LPS induction significantly increased the total cell count and the numbers of neutrophils and macrophages in the BALF (*p* < 0.01). Notably, intervention with Nag C liposomes significantly ameliorated the aforementioned inflammatory cell infiltration. Compared with those in the model group, the numbers of neutrophils and macrophages in the BALF in the low-dose treatment group were significantly lower (*p* < 0.05). In contrast, the medium- and high-dose treatment groups presented marked decreases in total cell numbers, as well as neutrophil and macrophage counts (*p* < 0.01), with the high-dose group demonstrating the most pronounced attenuation of inflammatory cell infiltration. These data confirmed that Nag C liposomes possessed dose-dependent anti-inflammatory activity (Figure 4E–G).

Optical microscopy revealed intact alveolar architecture with uniform septal spacing and no evident inflammatory infiltration or congestion in the lungs of the mice in the control group. Histopathological examination of LPS-induced model mice at 24 h post-exposure revealed characteristic features of ALI, including markedly widened alveolar septa, disruption of alveolar walls, diffuse inflammatory cell infiltration (predominantly neutrophils and macrophages), and pronounced congestion and edema in both interstitial and alveolar spaces. Histological analysis revealed significantly attenuated alveolar structural damage and markedly reduced inflammatory cell infiltration in the DXMS (positive control) group compared with the model group, confirming both successful model establishment and therapeutic efficacy of the intervention. Following treatment with Nag C liposomes, the low-dose group showed partial alleviation of alveolar septal thickening with residual focal inflammatory infiltration, whereas the high-dose group exhibited near-normal pulmonary histoarchitecture with only minimal inflammatory cells and essentially resolved edema. These pathological improvements were clearly dose-dependent. These findings demonstrated that Nag C liposomes can significantly ameliorate pulmonary histopathological damage in a dose-dependent manner (Figure 4H).

Myeloperoxidase (MPO), a specific marker of neutrophil activation, directly reflects the extent of neutrophil infiltration in tissues and serves as a crucial indicator for assessing inflammatory severity [36]. In this study, MPO expression in lung tissues was detected via an immunofluorescence assay. The results revealed a significant increase in fluorescence intensity in the lung tissues of the model group compared with those of the control group, with abundant MPO-positive cells predominantly localized in the alveolar septa and peribronchial regions. Compared with the positive control, Nag C liposomes significantly reduced the MPO-positive cell area, with the high-dose group showing no statistically significant difference in the MPO-positive area. These results indicated that Nag C liposomes dose-dependently suppress MPO expression in lung tissues, suggesting their potential to alleviate pulmonary inflammatory responses by suppressing neutrophil infiltration (Figure 4I).

**Figure 5:**
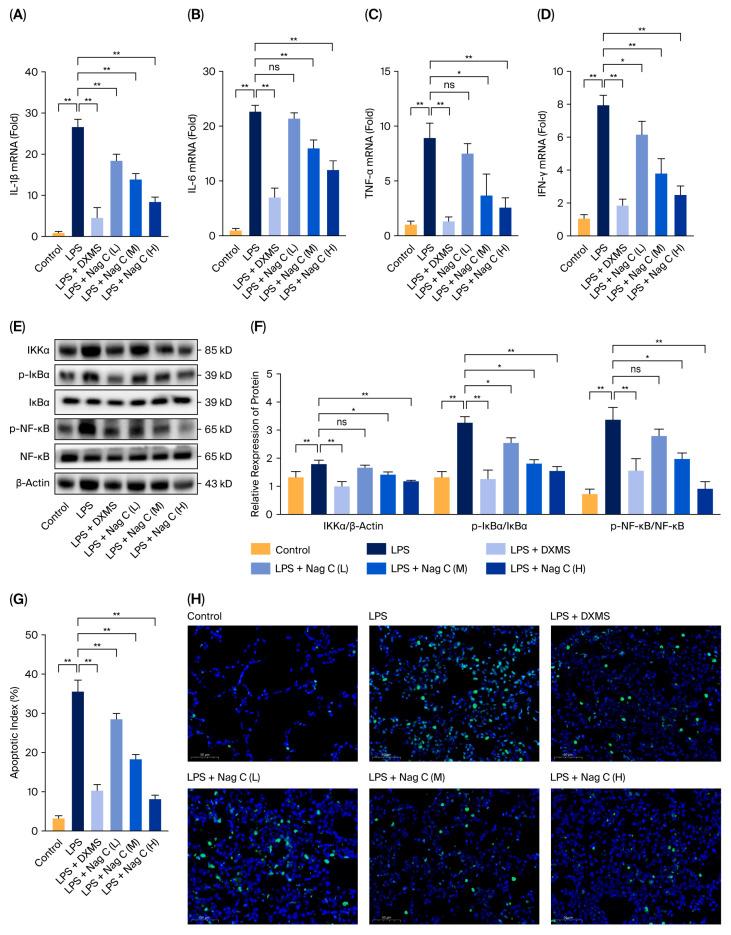
Effects of Nag C liposomes on inflammation and apoptosis in the lung tissues of mice with LPS-induced ALI. ((**A**–**D**). qRT–PCR analysis of the mRNA expression levels of inflammatory factors in the lung tissue of the mice (*n* = 3). (**E**,**F**). Western blot analysis of NF-κB pathway-related protein expression and phosphorylation levels in mouse lung tissue (*n* = 3). (**G**). Apoptosis index in mouse lung tissue (*n* = 3). (**H**). Apoptosis level in mouse lung tissue; scale bar: 50 μm. The above data are expressed as the means ± SDs. *, *p* < 0.05, **, *p* < 0.01, ns = not significant vs. cells in the model group).

The mRNA expression levels of the pro-inflammatory cytokines IL-1β, IL-6, TNF-α, and IFN-γ in lung tissues were quantitatively analyzed via real-time fluorescent quantitative PCR (RT–qPCR). The results demonstrated significantly upregulated expression of inflammatory factors in the LPS-induced model group compared with the control group (*p* < 0.01), confirming successful establishment of the inflammatory model. The positive control DXMS significantly attenuated the LPS-induced upregulation of inflammatory cytokines, demonstrating the therapeutic potential of this experimental model. Compared with LPS (in the model group), Nag C liposomes significantly decreased the levels of IL-1β and IFN-γ in the low-dose group (*p* < 0.05), whereas no significant changes in the IL-6 and TNF-α levels were detected (*p* > 0.05). Furthermore, both the medium- and high-dose groups presented significantly lower mRNA expression levels of IL-1β, IL-6, TNF-α, and IFN-γ than did the LPS model group (*p* < 0.01), with the most pronounced reduction observed in the high-dose group. These results demonstrated that Nag C liposomes effectively suppressed the transcriptional response of pro-inflammatory factors in lung tissue in a dose-dependent manner (Figure 5A–D).

The NF-κB signaling pathway serves as a pivotal transcription factor in regulating inflammatory responses and plays a critical role in inflammatory diseases such as ALI [37]. Upon cellular stimulation, the IκB kinase (IKK) complex is activated, leading to phosphorylation and subsequent degradation of the IκBα protein. This process results in the release and nuclear translocation of the NF-κB p65 subunit, thereby promoting the transcription of pro-inflammatory cytokines such as IL-1β, IL-6, and TNF-α [38]. This study investigated the regulatory effects of Nag C liposomes on key proteins of the NF-κB signaling pathway using Western blot analysis. Compared with the control group, the LPS model group presented significantly increased expression levels of IKKα and phosphorylation levels of IκBα and NF-κB in lung tissues, indicating successful activation of the NF-κB signaling pathway in the inflammatory model. Compared with treatment with LPS (in the model group), low-dose Nag C liposome treatment significantly attenuated the phosphorylation of both IκBα and NF-κB (*p* < 0.01). The high-dose group demonstrated more pronounced inhibitory effects, effectively restoring both IKKα expression levels and IκBα/NF-κB phosphorylation to baseline levels comparable to those of the normal control group, with efficacy equivalent to that of the positive control group. These results indicated that Nag C liposomes dose-dependently inhibited the activation of the NF-κB signaling pathway (Figure 5E,F).

This study quantitatively analyzed apoptosis in lung tissues via a TdT-mediated dUTP nick-end labeling (TUNEL) assay combined with 4′,6-diamidino-2-phenylindole (DAPI) staining. Compared with the control group, the model group presented a significant increase in the proportion of TUNEL-positive cells, with TUNEL staining cells predominantly colocalized with staining of alveolar epithelial cell markers. Treatment with Nag C liposomes significantly reduced the number of apoptotic cells. Notably, the high-dose group demonstrated more pronounced antiapoptotic effects that restored the positive rate to near-normal levels. The results indicated that Nag C liposomes attenuate inflammation-induced apoptosis in lung tissues in a distinct dose-dependent manner (Figure 5G).

### 2.5. Differential Gene Expression Profiling by High-Throughput RNA Sequencing

**Figure 6:**
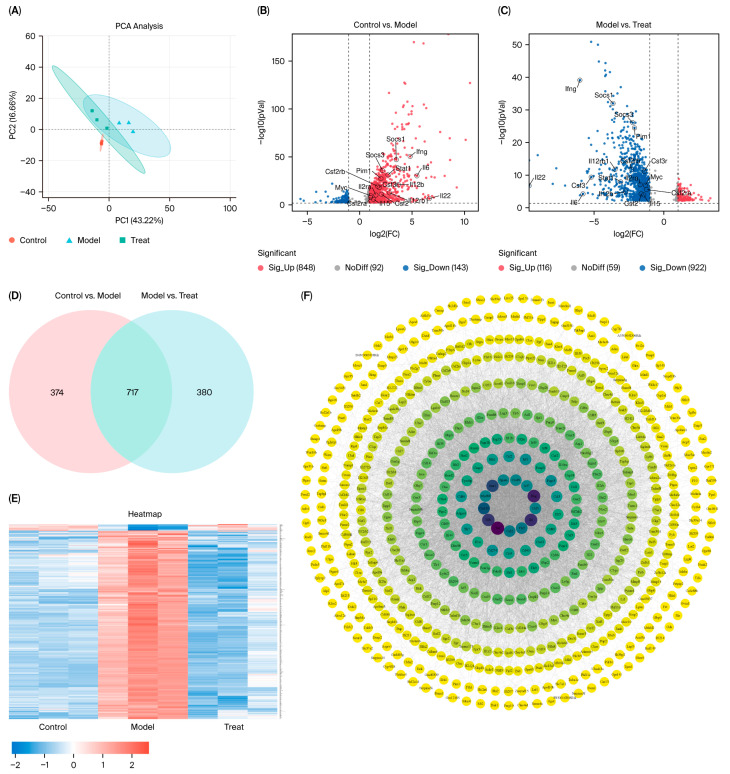
Sample quality control and interactive analysis of intergroup differentially expressed genes. ((**A**). Intergroup PCA. (**B**,**C**). Volcano plot analysis of intergroup differentially expressed genes. (**D**). Venn diagram. (**E**). Heatmap of differentially expressed genes (DEGs). (**F**). PPI network of intersecting genes identified via Venn diagram analysis).

As shown in Figure 6A, all experimental groups exhibited low dispersion (tight clustering of data points) in the PC1 and PC2 space, indicating high reproducibility among biological replicates within each group. The experimental treatment did not induce significant reconfiguration of gene expression patterns but resulted in good intragroup reproducibility and high data quality. The samples from the treatment group were clustered near those from the control group (e.g., with a small intercluster centroid distance), suggesting that the gene expression profiles under the treatment conditions were highly similar to those under the control conditions. This may indicate either a weak treatment effect or a time-dependent delay in the transcriptional response.

Volcano plot analysis (Figure 6B,C) revealed 991 significant differentially expressed genes (DEGs) between the control and model groups, including 848 upregulated genes and 143 downregulated genes. Comparative analysis between the model and treatment groups revealed 1038 DEGs, comprising 116 upregulated genes and 922 downregulated genes. Further Venn diagram analysis of the DEGs between the control vs. model and model vs. treatment groups (Figure 6D) revealed 717 coregulated DEGs, suggesting that these genes may represent key functional gene clusters that mediate both the biological effects of model group treatment and the therapeutic response to treatment group intervention. As demonstrated in Figure 6E, the majority of genes were upregulated in the model group compared with the control group, whereas the treatment group intervention resulted in a marked downregulation of these genes. The 717 shared target genes were imported into the STRING database to construct a protein–protein interaction (PPI) network. The resulting network comprised 641 nodes and 9829 edges, with an average node degree of 30.7 and an average betweenness centrality of 0.509. The core hub targets within the central network region, including inflammatory response-related genes (e.g., Stat1, Ifng, Ccl3, Il1b, and Il6), exhibited the highest interaction connectivity (Figure 6F).

**Figure 7:**
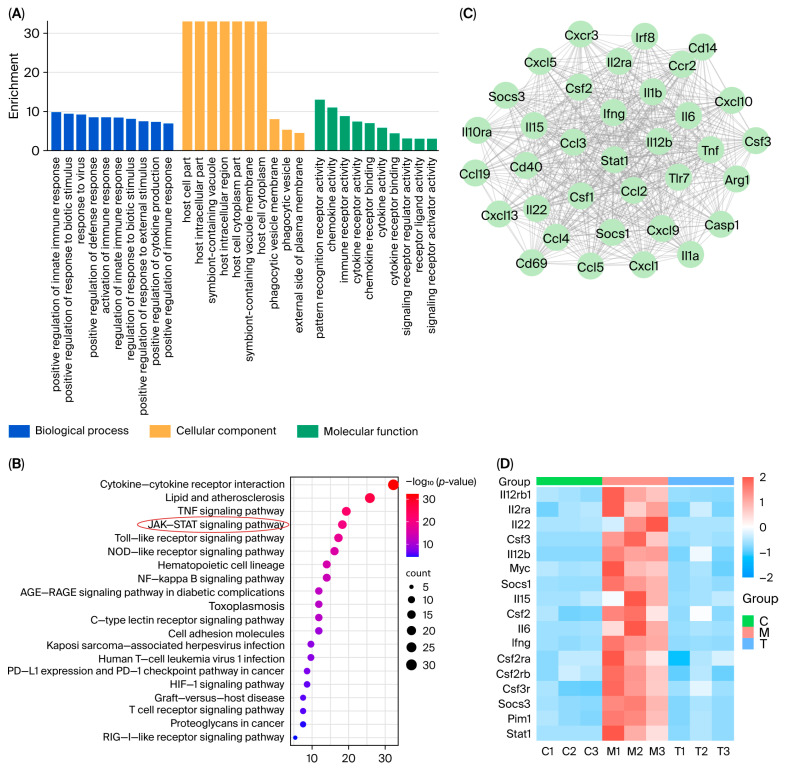
Signaling pathway analysis of the DEGs. ((**A**). GO enrichment analysis. (**B**). KEGG enrichment analysis. (**C**). Cluster analysis of the PPI network: highly connected subnetwork. (**D**). Heatmap of JAK–STAT-related gene expression profiles).

GO enrichment analysis of the 717 shared DEGs was performed using the Metascape database (Figure 7A). The analysis revealed significant enrichment of the pathways, including the immune system process, regulation of the immune system process, and defense response, all of which are closely associated with immune-inflammatory responses. Subsequent Kyoto Encyclopedia of Genes and Genomes (KEGG) pathway enrichment analysis of genes involved in immune system processes revealed significant enrichment of immune-inflammatory response-related genes in the JAK–STAT signaling pathway (Figure 7B). These findings collectively demonstrated that both the model group’s pathological effects and the treatment group’s therapeutic effects were predominantly mediated through the JAK–STAT signaling pathway. Further cluster analysis of the PPI network revealed highly interconnected subnetworks, including an inflammation-associated target module centered on Stat1 (Figure 7C), suggesting a potentially pivotal role of the JAK–STAT signaling pathway in inflammatory initiation and progression. The transcriptomic profiles of the JAK–STAT signaling pathway-associated DEGs were visualized using a heatmap (Figure 7D).

### 2.6. Nag C Ameliorates LPS-Induced Inflammation in RAW264.7 Cells via the STAT Signaling Pathway

**Figure 8:**
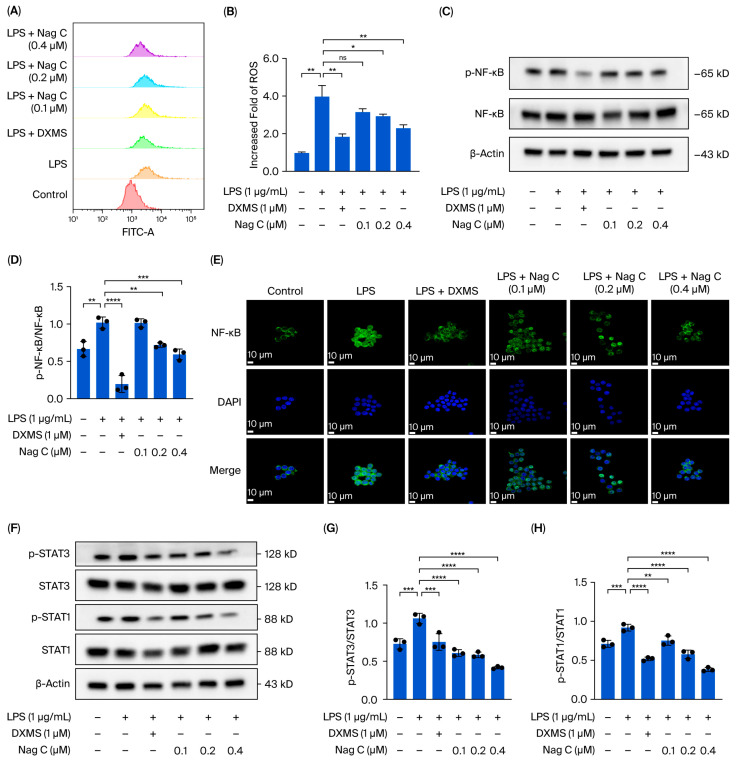
Protective effect of Nag C against LPS-induced inflammatory injury in RAW264.7 cells and its involvement in the STAT signaling pathway. ((**A**,**B**). ROS levels in RAW264.7 cells (*n* = 3). (**C**,**D**). Western blot analysis of NF-κB protein expression and phosphorylation status. (**E**). Immunofluorescence detection of NF-κB nuclear translocation; scale bar: 10 μm (*n* = 3). (**F**–**H**). Western blotting analysis of the expression of proteins associated with the STAT signaling pathway (*n* = 3). The above data are expressed as the means ± SDs. *, *p* < 0.05, **, *p* < 0.01, ***, *p* < 0.001, ****, *p* < 0.0001, ns = not significant vs. cells in the model group).

The antioxidant capacity of Nag C was investigated by measuring ROS levels. Compared with those in the control group, the ROS levels in the LPS stimulation group were significantly greater (*p* < 0.01). Compared with those in the model group, the ROS levels in the DXMS-treated group were significantly lower (*p* < 0.01), indicating the stability and reliability of the experimental system. Compared with those in the LPS model group, the ROS levels in the Nag C groups were significantly reduced. Particularly prominent effects were observed in the medium- and high-dose groups (*p* < 0.05 and *p* < 0.01, respectively) (Figure 8A,B).

To further investigate the mechanism by which Nag C regulates inflammatory signaling pathways, the expression and phosphorylation status of key proteins in the NF-κB signaling pathway were systematically analyzed via Western blot analysis. The results demonstrated that LPS stimulation significantly activated the NF-κB signaling pathway, as evidenced by a marked increase in NF-κB phosphorylation levels compared with those in the control group (*p* < 0.01). Nag C treatment significantly suppressed activation of this pathway in a dose-dependent manner. Moreover, total NF-κB protein expression did not significantly differ among the groups (*p* > 0.05), indicating that Nag C exerts its anti-inflammatory effects primarily through regulating NF-κB phosphorylation rather than affecting its protein synthesis. These results molecularly demonstrated that Nag C alleviates inflammatory responses by specifically inhibiting hyperactivation of the NF-κB signaling pathway (Figure 8C,D).

Additionally, immunofluorescence analysis was employed to evaluate Nag C’s ability to modulate NF-κB nuclear translocation dynamics. The results revealed a significant increase in nuclear fluorescence intensity following LPS induction. Following Nag C treatment, varying degrees of reduction in nuclear fluorescence intensity were observed, with the high-dose group exhibiting nearly complete restoration to control levels (Figure 8E).

Transcriptomic analysis revealed that the therapeutic effects of Nag C on ALI were associated primarily with the JAK–STAT signaling pathway. To further validate this finding, we performed Western blot analysis to examine the expression and phosphorylation status of the key pathway proteins STAT1 and STAT3. The results demonstrated that LPS induction significantly increased the levels of both p-STAT1 and p-STAT3. Following treatment with Nag C, both p-STAT1 and p-STAT3 levels were significantly reduced, with STAT1 demonstrating more pronounced dephosphorylation. This inhibitory effect was clearly dose-dependent. These results indicated that the anti-inflammatory effects of Nag C were associated with the JAK–STAT signaling pathway, particularly the stronger correlation with the STAT1 signaling axis (Figure 8F–H).

### 2.7. The Inhibitory Effect of Nag C on the STAT Pathway Was Further Validated via the Use of Specific STAT Pathway Inhibitors

**Figure 9:**
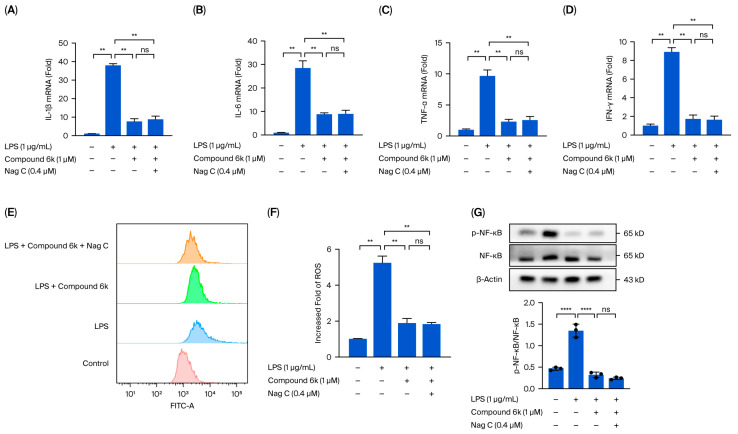
Protective effects of Nag C on LPS-induced inflammation in RAW264.7 macrophages post-STAT pathway blockade. ((**A**–**D**). Effects of Nag C on LPS-induced inflammation in RAW264.7 cells following STAT pathway blockade (*n* = 3). (**E**,**F**). Flow cytometric analysis of intracellular ROS production (*n* = 3). (**G**). Western blot analysis of NF-κB protein expression and phosphorylation status (*n* = 3). The above data are expressed as the means ± SDs. **, *p* < 0.01, ****, *p* < 0.0001, ns = not significant vs. cells in the model group).

This study demonstrated that the therapeutic effects of Nag C against LPS-induced inflammation are mediated through the STAT signaling pathway. However, whether Nag C exerts its anti-inflammatory effects through the inhibition of the STAT signaling pathway requires further experimental validation. STAT1/STAT3 inhibitors (STAT1/3-IN-1, Compound 6k) were employed to pharmacologically block the STAT signaling pathway in RAW264.7 cells in vitro. Following LPS-induced inflammatory stimulation, we investigated whether Nag C retained its therapeutic efficacy against cellular inflammation after STAT pathway inhibition. The results demonstrated that pharmacological blockade of the STAT pathway with Compound 6k significantly reduced the LPS-induced production of pro-inflammatory cytokines (IL-6, IL-1β, TNF-α, and IFN-γ) in RAW264.7 cells compared with that in the LPS group (*p* < 0.01). These findings demonstrated that the STAT signaling pathway plays a critical role in inflammatory pathogenesis. Targeted inhibition of this pathway may represent an effective therapeutic strategy against inflammation. Compared with the LPS + Compound 6k group, the Nag C treatment group presented no significant difference in the levels of inflammatory cytokines (IL-6, IL-1β, TNF-α, and IFN-γ) following STAT pathway blockade (*p* > 0.05). These findings indicated that Nag C’s anti-inflammatory effects were abolished upon STAT inhibition, further confirming that its therapeutic action primarily depends on the suppression of this signaling pathway (Figure 9A–D).

ROS detection revealed that pharmacological blockade of the STAT pathway with Compound 6k significantly reduced LPS-induced ROS levels in RAW264.7 cells compared with those in the LPS group (*p* < 0.01). These findings indicate that inhibition of the STAT signaling pathway can effectively mitigate inflammation-associated oxidative stress damage. Compared with the LPS + Compound 6k group, the Nag C treatment group presented no significant difference in ROS levels after STAT pathway blockade with Compound 6k (*p* > 0.05). These results indicate that the antioxidant effects of Nag C were significantly attenuated upon STAT pathway blockade, providing conclusive evidence that its anti-inflammatory efficacy is mediated primarily through suppression of this signaling pathway (Figure 9E,F).

The protein expression and phosphorylation status of NF-κB in RAW264.7 cells were analyzed by Western blotting. Following STAT pathway blockade with Compound 6k, Nag C treatment resulted in no significant difference in NF-κB phosphorylation compared with that in the LPS + Compound 6k group (*p* > 0.05). These results demonstrate that the STAT signaling pathway plays a pivotal role in mediating the anti-inflammatory effects of Nag C (Figure 9G).

## 3. Discussion

ALI is a life-threatening clinical syndrome caused by various etiological factors and is characterized by pathological features, including inflammatory cell infiltration, pulmonary edema, and alveolar epithelial apoptosis [2]. ALI is pathologically characterized by the infiltration of inflammatory cells and an increase in pro-inflammatory mediators, leading to disruption of the alveolar–capillary barrier and pulmonary edema, which ultimately results in severe impairment of gas exchange [1,9]. Hence, modulating inflammation is pivotal for preventing ALI progression to ARDS.

Natural products serve as reliable sources of anti-inflammatory agents [39,40]. Numerous natural products, e.g., flavonoids, alkaloids, and polyphenols, have been reported to exert protective effects against ALI [41]. These compounds exert therapeutic effects against ALI through multiple mechanisms, including inhibition of classical inflammatory pathways (e.g., NF-κB and MAPK signaling), suppression of neutrophil infiltration, promotion of macrophage polarization toward anti-inflammatory phenotypes, and upregulation of tight junction proteins to maintain epithelial barrier integrity [42]. Additionally, many natural products exert cytoprotective effects by suppressing mitochondrial apoptotic pathways, scavenging oxygen free radicals, and promoting cellular damage repair, which also contributes to the management of ALI [43]. Terpenoids are recognized as a class of highly promising natural anti-inflammatory agents because of their broad natural occurrence, structural diversity, and pronounced anti-inflammatory effects. Studies have revealed that terpenoids exert anti-inflammatory effects through multiple mechanisms, including the suppression of inflammatory mediator production (e.g., prostaglandins, leukotrienes, and cytokines), the modulation of inflammation-related signaling pathways (e.g., the NF-κB, MAPK, and JAK–STAT pathways), and the inhibition of inflammatory cell activation [44,45,46]. These findings provide crucial scientific evidence for further research and development of terpenoid-based therapeutics.

In this study, the chemical constituents of the seeds of *P*. *nakaii*, a terpenoid-rich material, were investigated, leading to the isolation and characterization of twelve compounds (**1**–**12**). Preliminary screening revealed that Nag C exhibited potent anti-inflammatory activity. The anti-inflammatory mechanisms of Nag C were systematically investigated in an LPS-induced murine ALI model. To address the poor solubility of Nag C and increase its bioavailability and therapeutic efficacy against ALI, Nag C liposomes were prepared for inhalation administration. The therapeutic potential of this inhalable formulation in ALI treatment was systematically evaluated. The results demonstrated that the Nag C liposome formulation significantly ameliorated LPS-induced histopathological damage in lung tissues, reduced neutrophil infiltration and pro-inflammatory cytokine levels, and increased the levels of key biomarkers, including myeloperoxidase (MPO) activity and apoptotic indices. Mechanistically, Nag C exerts therapeutic effects on ALI via the modulation of the canonical NF-κB pathway. The findings of this study provide experimental evidence to support the development of Nag C as a therapeutic agent.

This study focused on the systematic mechanistic investigation of Nag C for the treatment of ALI. Initial transcriptomic profiling and analysis of lung tissues from Nag C-treated mice revealed the significant involvement of the JAK–STAT signaling pathway. To validate the hypothesis that Nag C exerts its anti-inflammatory effects via STAT pathway inhibition, we first examined its ability to modulate LPS-induced inflammation in RAW264.7 macrophages in relation to STAT signaling. These results demonstrated that Nag C concurrently suppressed inflammatory responses and significantly downregulated the phosphorylation levels of the NF-κB, STAT3, and STAT1 proteins, supporting its crucial involvement in the modulation of the STAT signaling pathway. Subsequent pharmacological inhibition of STAT3/STAT1 in RAW264.7 cells significantly reduced inflammatory cytokine levels, ROS levels, and NF-κB activity, confirming that targeted STAT blockade is a viable anti-inflammatory strategy. Notably, when Nag C was administered following STAT3/STAT1 inhibition, no additional suppression of these inflammatory markers was observed, providing compelling evidence that Nag C exerted its anti-inflammatory effects primarily through STAT pathway modulation. Finally, assessment of STAT pathway activation in murine lung tissues demonstrated that Nag C similarly suppressed the phosphorylation of STAT3 and STAT1 proteins in vivo, confirming that STAT pathway inhibition is the mechanistic basis for the therapeutic efficacy of Nag C against ALI.

This study revealed that Nag C is a bioactive component with a protective effect against ALI from the seeds of *P. nakaii*. Mechanistic experiments demonstrated that Nag C exerts its therapeutic effects by inhibiting the STAT signaling pathway. These results highlight a promising lead compound in the development of novel anti-inflammatory therapeutics targeting this pathway. Notably, this study pioneers the association between STAT3/STAT1 dual-inhibition strategies and the anti-inflammatory mechanisms of natural products. These findings not only advance the understanding of terpenoid pharmacological activities but also establish an innovative therapeutic paradigm for precise modulation of STAT signaling networks.

## 4. Materials and Methods

### 4.1. Reagents

RAW264.7 cells were obtained from Meilun Biotechnology Co., Ltd. (Dalian, China). High-glucose DMEM and trypsin were purchased from Zhejiang Senrui Biotechnology Co., Ltd. (Huzhou, China). Serum-free cell freezing medium was purchased from Wuhan San Ying Biotechnology Co., Ltd. (Wuhan, China). Phosphate-buffered saline (PBS), hematoxylin and eosin (H&E) stain, and neutral balsam were purchased from Beijing Solarbio Science & Technology Co., Ltd. (Beijing, China). Thiazolyl blue tetrazolium bromide (MTT) was purchased from Shanghai Beyotime Biotechnology Co., Ltd. (Shanghai, China). TRIzol reagent was purchased from Tiangen Biotech (Beijing) Co., Ltd. (Beijing, China). An RNA reverse transcription kit was purchased from Takara Bio, Inc. (Kusatsu, Shiga, Japan). Quantitative real-time PCR (qPCR) reagents were purchased from Yeasen Biotechnology (Shanghai) Co., Ltd. (Shanghai, China). A TUNEL assay kit was purchased from Shanghai Aladdin Biochemical Technology Co., Ltd. (Shanghai, China). The protease inhibitor, phosphatase inhibitor, loading buffer, bicinchoninic acid (BCA) protein assay kit, bovine serum albumin (BSA), and enhanced chemiluminescence (ECL) detection reagents were purchased from Shanghai Beyotime Biotechnology Co., Ltd. (Shanghai, China). DAB chromogenic solution was purchased from Shanghai Aladdin Biochemical Technology Co., Ltd. (Shanghai, China). The protein molecular weight marker was purchased from Absin Bioscience, Inc. (Shanghai, China). The 12% precast polyacrylamide gels and antibody dilution buffer were purchased from Yeasen Biotechnology Co., Ltd. (Shanghai, China). The polyvinylidene fluoride (PVDF) membrane was purchased from Merck KGaA (Darmstadt, Germany). Antibodies against NF-κB (8242S), p-NF-κB (3039S), IκBα (4812S), p-IκBα (2859S), STAT3 (4113T), p-STAT3 (9139T), and β-actin (4967S) were obtained from Cell Signaling Technology Co., Ltd. (Waltham, MA, USA). Anti-STAT1 (ab31369), anti-p-STAT1 (ab109461), and antimyeloperoxidase (MPO) (ab90810) primary antibodies were purchased from Abcam (Waltham, MA, USA). Horseradish peroxidase (HRP)-conjugated goat anti-rabbit secondary antibody, Alexa Fluor 488-conjugated goat anti-rabbit secondary antibody, and Alexa Fluor 647-conjugated goat anti-mouse IgG (H + L) secondary antibody were purchased from Shanghai Beyotime Biotechnology Co., Ltd. (Shanghai, China).

All the solvents used for chromatography were purchased from Sinopharm Chemical Reagent Co., Ltd. (Shanghai, China). TLC plates (GF254) and silica gel (200–300 mesh) were purchased from Qingdao Haiyang Chemical Factory (Qingdao, Shandong, China). MCI CHP20P resin (75–150 μm) was purchased from Mitsubishi Chemical Corporation (Tokyo, Japan). The ODS-C18 gel (50 μm) was purchased from YMC Co., Ltd. (Kyoto, Japan). Chromatography gels (HW40 and HW60) were purchased from TOSOH Corporation (Tokyo, Japan).

### 4.2. Plant Material

The plant material was collected from Wuxi, Jiangsu Province, China, in October 2017. They were identified as seeds of *P. nakaii* by Prof. Yong-Hong Zhang from Fujian Medical University, China. A sample (No. P20171008) was deposited at Zhejiang University of Technology.

### 4.3. Cells

RAW264.7 cells (mouse monocyte/macrophage leukemia cells) were obtained from Meilun Biotechnology Co., Ltd. (Dalian, China).

### 4.4. Animals

Eight-week-old male C57BL/6 mice were obtained from the Experimental Animal Center of Hangzhou Medical College (Hangzhou, Zhejiang, China) and maintained in specific pathogen-free (SPF) facilities. The housing conditions included a temperature range of 20–26 °C, a relative humidity of 40–70%, and a 12 h light/dark cycle. All animal experiments and procedures were conducted in compliance with the international standards of the Association for Assessment and Accreditation of Laboratory Animal Care (AAALAC) accreditation program and were reviewed and approved by the Animal Ethics Committee of Hangzhou Medical College (Approval No. 2021–029).

### 4.5. Extraction and Isolation of Compounds ***1***–***12***

Dry seeds of *P. nakaii* (2.5 kg) were ground into a coarse powder and extracted by reflux with 70% ethanol (3 times, 2 h each). The ethanol extract (316 g) was then partitioned between chloroform and water. The resulting chloroform-soluble residue (66 g) was subjected to silica gel column chromatography (CC) (CHCl_3_/MeOH 20:1 → 0:1, *v*/*v*) to obtain six fractions, Fr.1–Fr.6. Fr. 2 (1.1 g) was purified by MCI gel CC (MeOH/H_2_O 20:80 → 90:10, *v*/*v*) to yield three subfractions, 2A–2C. Fr. 2A was purified by silica gel CC (petroleum ether/acetone 8:1 → 3:1, *v*/*v*) to afford 7 (22.1 mg). Fr. 3 (2.3 g) was fractionated into five fractions, 3A–3E, by CC on MCI gel (MeOH/H_2_O 20:80 → 90:10, *v*/*v*). Fr. 3A was purified by silica gel CC (petroleum ether/acetone 8:1 → 5:1, *v*/*v*) to yield 9 (5.1 mg). Fr. 3C was purified using an LH-20 gel (MeOH) to afford 10 (2.0 mg). Fr. 3D was purified by ODS C18 CC (MeOH/H_2_O 50:50 → 70:10, *v*/*v*) to produce 11 (2.1 mg). Fr. 5 (5.8 g) was first divided into eight fractions, 5A–5F, by MCI gel CC (MeOH/H_2_O 50:50 → 90:10, *v*/*v*). Compound **3** (307.4 mg) was obtained by crystallization from Fr. 5A. Compound **8** (2.5 mg) was obtained from Fr. 5B by silica gel CC (CHCl_3_/MeOH 20:1, *v*/*v*). Fr. 5D was purified by silica gel CC (CHCl_3_/MeOH 20:1 → 15:1, *v*/*v*) to yield 4 (20.2 mg) and 5 (3.2 mg). Fr. 5E was separated by silica gel CC (CHCl_3_/MeOH (80:1 → 60:1, *v*/*v*)) to afford 1 (30.8 mg) and 2 (13.6 mg). Compound **6** (99.8 mg) was obtained from Fr. 5F by silica gel CC (CHCl_3_/MeOH (60:1 → 20:1, *v*/*v*)). Fr. 5H was separated by CC on silica gel (petroleum ether/acetone (8:1 → 3:1, *v*/*v*) to give 12 (7.1 mg). The structures of compound **1** were confirmed via spectroscopic methods (^1^H-NMR, ^13^C-NMR, DEPT, HMBC, ^1^H-^1^H-COSY, NOESY, HSQC, HRESIMS, and single-crystal X-ray diffraction (**Cu-Kα**)). Compounds **2**–**12** were identified by comparison of the ^1^H- and ^13^C-NMR data with those reported in the literature.

### 4.6. In Vitro Anti-Inflammatory Activity Evaluation of Compounds ***1***–***12***

The effects of compounds **1**–**12** on the viability of RAW264.7 cells were initially assessed via the MTT method. RAW264.7 cells in the logarithmic growth phase were selected, trypsinized with 0.25% trypsin, and prepared as single-cell suspensions. The suspension was evenly seeded into a 96-well plate (100 μL per well) and incubated at 37 °C in a humidified atmosphere with 5% CO_2_ for 12 h to allow partial adhesion. Following incubation, the original culture medium was removed and replaced with experimental medium containing compounds **1**–**12** at concentrations ranging from 0.097 to 50 μM. The final concentration of dimethyl sulfoxide (DMSO) used was 0.1‰ (*v*/*v*) in all the treatment groups. A control group containing only 0.1‰ DMSO was included. Each experimental condition was assayed in quintuplicate to minimize experimental error. The absorbance (OD value) of each well was measured at a wavelength of 490 nm using a microplate reader after 48 h, and the IC_50_ was calculated.

For the anti-inflammatory assay, RAW264.7 cells were allocated into six groups, namely, the control group (DMSO, 1‰), the LPS-induced model group, the dexamethasone (DXMS)-positive control (LPS + 50 μg/mL DXMS), and the treatment groups (high-dose, medium-dose, and low-dose). All groups except the control group were stimulated with LPS at a final concentration of 1 μg/mL. After 2 h of LPS induction, the treatment groups were exposed to compounds **1**–**12**. The positive control received DXMS at a final concentration of 50 μg/mL. Following 12 h of treatment, the cells from all the groups were harvested for subsequent analyses. Total RNA was extracted from cells using TRIzol reagent and subsequently reverse-transcribed into cDNA using a reverse transcription kit. The cDNA was then subjected to RT–qPCR analysis using a fluorescent quantitative PCR reagent. The expression levels of the inflammatory factors IL-1β, IL-6, and TNF-α were determined; GAPDH served as the internal reference gene.

### 4.7. Preparation of Nag C Liposomes

Initially, 4 mg of Nag C, 40 mg of soybean phospholipid (drug-to-lipid ratio = 1:10), and 8 mg of cholesterol (phospholipid-to-cholesterol ratio = 5:1) were weighed and dissolved in 2 mL of anhydrous ethanol, followed by sonication until a clear solution was obtained. The solution was then slowly and uniformly dripped into 10 mL of phosphate-buffered saline (PBS) under magnetic stirring at 45 °C. Finally, the ethanol was evaporated to obtain Nag C liposomes. The physicochemical properties of Nag C liposomes were characterized. The average diameter was determined by transmission electron microscopy (TEM), whereas the zeta potential and encapsulation efficiency were quantified using dynamic light scattering (DLS) and an ultrafiltration/HPLC method, respectively. Then encapsulation efficiency was analyzed on the prepared Nag C liposomes formulation following one week of storage at 4 °C under controlled conditions.

### 4.8. Animals and Experimental Protocol

Forty-eight male C57BL/6 mice (8 weeks old) were randomly divided into six groups (*n* = 8 per group): the control group (PBS only), the LPS model group (LPS alone), the DXMS group (LPS + DXMS (10 mg/kg)), the Nag C low-dose (L) group (LPS + Nag C (0.25 mg/kg)), the Nag C medium-dose (M) group (LPS + Nag C (0.5 mg/kg)), and the Nag C high-dose (H) group (LPS + Nag C (1 mg/kg)). After a 2-day acclimatization period, the mice in the Nag C treatment groups received the corresponding doses via intranasal inhalation (50 μL per administration, twice daily). Twenty-four hours post-treatment, all groups except the control group were anesthetized with 1% sodium pentobarbital (0.1 mL per mouse) and subsequently administered 50 μL of LPS (20 μg per mouse) via intranasal instillation. Four hours after LPS induction, the DXMS group received an intraperitoneal injection of dexamethasone (10 mg/kg), while the Nag C treatment groups were administered their respective doses via intranasal inhalation. The control group received PBS only through intranasal instillation. Twenty-four hours after LPS induction, the mice were euthanized, and samples were collected for further analysis.

### 4.9. Body Weight and Lung Index Measurements

Body weights were recorded daily throughout this study to monitor changes. At the experimental endpoint, eight mice per group were randomly selected using a random number table. The selected animals were anesthetized with 1% sodium pentobarbital and euthanized via orbital exsanguination, followed by necropsy. Harvested lung tissues were immediately processed as follows: they were immersed in ice-cold normal saline (4 °C), rinsed three times to remove residual blood, and gently blotted on filter paper to remove surface moisture. Wet weights were recorded, and the organ coefficient (lung weight/body weight ratio) was calculated to evaluate pulmonary edema.

### 4.10. Collection and Processing of BALF

Three mice per group, selected randomly, were deeply anesthetized via intraperitoneal injection of 1% sodium pentobarbital. Following tracheal exposure through dissection, an intravenous indwelling needle was used for intubation. Lung lavage was performed by instilling and withdrawing 1 mL of ice-cold sterile saline (0.9% NaCl) three times. The collected BALF was immediately centrifuged (1500× *g*, 10 min, 4 °C). The supernatants were aliquoted into sterile microcentrifuge tubes and stored at −80 °C for subsequent analysis. The resulting cell pellet was resuspended in phosphate-buffered saline (PBS), and total cells, macrophages, and neutrophils were enumerated using a hemocytometer.

### 4.11. Real-Time Quantitative PCR

The PCR methodology is detailed in the referenced literature [47] and can be briefly described as follows. The tissue samples (20 mg) were weighed into prechilled nuclease-free microcentrifuge tubes and immediately homogenized in 1 mL of TRIzol reagent (Waltham, MA, USA) on ice until complete lysis was achieved. High-purity total RNA was isolated following the manufacturer’s protocol, including chloroform phase separation, isopropanol precipitation, and ethanol washing steps. The mRNA expression levels of pro-inflammatory cytokines (IL-1β, IL-6, and TNF-α) in the lung tissues were quantified via RT–qPCR as described in Section 4.6.

### 4.12. Western Blotting

Mouse lung tissues and RAW264.7 cells were placed in microcentrifuge tubes and homogenized in RIPA lysis buffer supplemented with protease and phosphatase inhibitors. The tissues were thoroughly disrupted on ice using a mechanical homogenizer, followed by a 20 min incubation on ice with intermittent vortexing every 5 min to ensure complete lysis. The lysates were subsequently centrifuged at 12,000× *g* for 15 min at 4 °C in a precooled centrifuge. The resulting supernatant was collected as the total protein extract. Subsequent procedures, including protein quantification, denaturation, SDS–PAGE, membrane transfer, antibody incubation, and chemiluminescent detection, were performed as previously described [47].

### 4.13. Histological and Imaging Tests of the Lungs

The lung tissue samples were collected from sacrificed mice, fixed with 4% paraformaldehyde for 24 h, embedded in paraffin, and sectioned at a thickness of 5 μm. The inflammatory status of the lung tissues was subsequently evaluated via HE staining, and MPO levels and apoptosis (TUNEL assay) were subsequently assessed via immunofluorescence analysis. The expression levels of Dectin-1 and Syk in lung tissues were determined using immunohistochemical analysis.

### 4.14. RNA Sequencing

Following a one-week acclimatization period, C57BL/6 mice were allocated into groups according to the experimental design detailed in Section 4.8. Upon completion of the experiment, lung tissues were collected from the mice in the control group, model control group (LPS-induced), and high-dose treatment group (*n* = 3 per group). The collected tissues were immediately snap-frozen in liquid nitrogen and subsequently stored at −80 °C. All samples were then transported under continuous cold chain conditions to the biobank for subsequent analysis. Initially, quality control was performed on the raw sequencing expression data obtained from mouse lung tissue samples. Principal component analysis (PCA) and differential transcriptome expression analysis were subsequently conducted. This analysis identified sets of DEGs for comparisons between the control and model groups and between the model and treatment groups. Finally, a Venn diagram was constructed to visualize the intersecting relationships among the DEG sets derived from these intergroup comparisons. Gene ontology (GO) enrichment analysis was subsequently conducted on the identified sets of DEGs. KEGG enrichment analysis was subsequently performed on the DEGs in the top-ranked (most significantly enriched) GO terms/pathways identified in the prior analysis. This comprehensive approach serves to investigate the functional roles and interactions of genes within metabolic pathways, signal transduction cascades, and disease-associated pathways, with the goal of identifying therapeutically relevant targets within these pathways. All target genes derived from the enrichment analyses were imported into the STRING database to construct a PPI network. The core targets within the network were subsequently predicted and visualized on the basis of their PPIs.

### 4.15. Flow Cytometry

The dichlorodihydrofluorescein diacetate (DCFH-DA) probe was diluted to a working concentration of 10 μM in serum-free medium, and all procedures were performed under light-protected conditions. Following aspiration of the culture media, the RAW264.7 macrophages in the 6-well plates were detached using gentle pipetting and centrifuged at 1000× *g* (4 °C, 5 min), and the resulting pellet was resuspended in 10 μM DCFH-DA working solution prepared in serum-free medium. The cells were incubated at 37 °C under 5% CO_2_ with light protection for 20 min and subjected to gentle agitation at 5 min intervals, followed by three washes with ice-cold PBS to remove insufficiently internalized probes after incubation. The cells were analyzed using the FITC channel on a flow cytometer, where the voltage and gain parameters were optimized to position the fluorescence signals within the linear detection range. The cellular fluorescence distribution was recorded. The flow cytometry data were analyzed using FlowJo software 10.8.1 to quantify the intracellular ROS levels.

### 4.16. Immunofluorescence

Adherent cells seeded in 6-well glass-bottom plates were centrifuged at 1000× *g* for 10 min. After media removal, the samples were dried at 30 °C for 2 h. The cells were then fixed with freshly prepared 4% paraformaldehyde for 15 min at room temperature (RT), followed by three 5 min washes with 0.1 M PBS buffer (pH 7.4). Permeabilization was performed with 0.5% Triton X-100 (Beijing Solarbio Science & Technology Co., Ltd., Beijing, China) for 15 min. Nonspecific binding was blocked with 10% goat serum for 1 h at RT prior to overnight incubation at 4 °C with a primary antibody against NF-κB p65 (1:200 dilution). After three 10 min PBST washes (with PBS containing 0.05% Tween-20), the samples were incubated with an Alexa Fluor 488-conjugated secondary antibody (1:500) for 1 h under light-protected conditions. The nuclei were counterstained with DAPI (1 μg/mL) for 5 min in darkness. Finally, the coverslips were mounted with ProLong Gold antifade reagent and imaged by confocal laser scanning microscopy.

### 4.17. Statistical Analysis

All data were obtained from at least three independent experiments and expressed as the mean ± standard deviation (mean ± SD). Statistical analysis was performed using SPSS 23.0. One-way analysis of variance (ANOVA) was used to assess intergroup differences, with a threshold of *p* < 0.05 considered statistically significant. Figures were generated using GraphPad Prism 10.0.

## 5. Conclusions

In this study, the major chemical constituents from the seed extract of *Podocarpus nakaii* were systematically isolated and characterized. The absolute configuration of podolactone E was unambiguously determined via single-crystal X-ray diffraction for the first time. Moreover, this study is the first to reveal that Nag C ameliorates ALI through selective inhibition of the STAT signaling pathway, representing the first mechanistic elucidation of this pharmacological activity. These findings provide rational guidance for pharmacological research and the clinical development of drugs derived from *Podocarpus* species and reveal the implications for their therapeutic applications.

## Data Availability

All data generated and analyzed during this study are included in this published article and its Supplementary Materials. The primary data supporting the findings of this study are openly available in the NCBI Sequence Read Archive (SRA) under accession number PRJNA1293737.

## Supplementary Materials

The following supporting information can be downloaded from https://www.mdpi.com/article/10.3390/ph18091319/s1, Spectroscopic data for compounds **1**–**12**. Figures S1–S30: NMR spectra of compounds **1**–**12**; Figures S31–S33: Cytotoxicity and anti-inflammatory assay of compounds **1**–**12**.
