# Supplementary material for: Nagilactone C from the Seeds of Podocarpus nakaii May Protect Against LPS-Induced Acute Lung Injury via STAT Signaling Pathway Inhibition
Source: Pharmaceuticals (Basel). 2025 Sep 3;18(9):1319. doi: 10.3390/ph18091319 (PMC12472992; doi:10.3390/ph18091319)
Supplement: Supplementary file 1 [file pharmaceuticals-18-01319-s001.zip › pharmaceuticals-3799833-supplementary.pdf]

Supplementary Materials

Content

- Spectroscopic data of compounds 1–20 2
- Figure S1. ^{1}H NMR spectrum of 1. 1
- Figure S2. ^{13}C NMR spectrum of 1. 1
- Figure S3. DEPT spectrum of 1.......... 2
- Figure S4. ^{1}H-^{1}H COSY spectrum of 1. 2
- Figure S5. HMBC spectrum of 1.......... 3
- Figure S6. HSQC spectrum of 1.......... 3
- Figure S7. NOESY spectrum of 1. 4
- Figure S8. HRESIMS of 1.......... 4
- Figure S9. ^{1}H NMR spectrum of 2. 5
- Figure S10. ^{13}C NMR spectrum of 2. 5
- Figure S11. ^{1}H NMR spectrum of 3. 6
- Figure S12. ^{13}C NMR spectrum of 3. 6
- Figure S13. ^{1}H NMR spectrum of 4. 7
- Figure S14. ^{13}C NMR spectrum of 4. 7
- Figure S15. ^{1}H NMR spectrum of 5. 8
- Figure S16. ^{13}C NMR spectrum of 5. 8
- Figure S17. ^{1}H NMR spectrum of 6. 9
- Figure S18. ^{13}C NMR spectrum of 6. 9
- Figure S19. ^{1}H NMR spectrum of 7. 10
- Figure S20. ^{13}C NMR spectrum of 7. 10
- Figure S21. ^{1}H NMR spectrum of 8. 11
- Figure S22. ^{13}C NMR spectrum of 8. 11
- Figure S23. ^{1}H NMR spectrum of 9. 12
- Figure S24. ^{13}C NMR spectrum of 9. 12
- Figure S25. ^{1}H NMR spectrum of 10. 13
- Figure S26. ^{13}C NMR spectrum of 10. 13
- Figure S27. ^{1}H NMR spectrum of 11. 14
- Figure S28. ^{13}C NMR spectrum of 11. 14
- Figure S29. ^{1}H NMR spectrum of 12. 15
- Figure S30. ^{13}C NMR spectrum of 12. 15
- Figure S31. Cytotoxicity testing and anti-inflammatory activity screening of 1–4. 16
- Figure S32. Cytotoxicity testing and anti-inflammatory activity screening of 5–8. 17
- Figure S33. Cytotoxicity testing and anti-inflammatory activity screening of 9–12. 18

Spectroscopic data of compounds 1–12

Podolactone E (1): Colorless needles. mp: 262–263 °C. $[\alpha]_D^{20}$ = –3.0 (*c* 0.1, $CHCl_3$); HR-ESI-MS (pos.) *m/z*: 331.1178 ([M + H]$^+$, $C_{18}H_{19}O_6^+$; Calcd. 331.1176). ^{1}H NMR (600 MHz, $CDCl_3$): δ_H 3.54 (1H, d, *J* = 4.0 Hz, H-1), 3.49 (1H, dd, *J* = 6.1, 4.0 Hz, H-2), 4.50 (1H, d, *J* = 6.1 Hz, H-3), 2.14 (1H, d, *J* = 5.0 Hz, H-5), 5.01 (1H, ddd, *J* = 6.2, 4.9, 1.6 Hz, H-6), 6.22 (1H, d, *J* = 1.6 Hz, H-7), 6.19 (1H, brs, H-11), 5.42 (1H, d, *J* = 7.6 Hz, H-14), 5.98 (1H, ddd, 17.2, 10.4, 7.6, H-15), 5.61 (1H, d, *J* = 10.4 Hz, H_a-16), 5.56 (1H, d, *J* = 17.2 Hz, H_b-16), 1.49 (3H, s, H_3-18), 1.27 (3H, s, H_3-20); ^{13}C NMR (150 MHz, $CDCl_3$): δ_C 55.7 (C-1), 50.3 (C-2), 68.3 (C-3), 48.4 (C-4), 49.2 (C-5), 71.4 (C-6), 122.7 (C-7), 135.2 (C-8), 155.8 (C-9), 36.6 (C-10), 113.4 (C-11), 163.0 (C-12), 80.8 (C-14), 131.5 (C-15), 123.1 (C-16), 25.5 (C-18), 178.1 (C-19), 19.2 (C-20).

Crystal Data for Podolactone E (**1**): $C_{18}H_{18}O_6$, M = 330.32 g/mol), monoclinic, space group P21 (no. 4), a = 6.8556(2) Å, b = 11.2085(3) Å, c = 10.1501(3) Å, β = 103.8670(10)°, V = 757.21(4) Å3, Z = 2, T = 291.02 K, μ(CuKα) = 0.912 mm-1, Dcalc = 1.449 g/cm^3, 22755 reflections measured (15.482° ≤ 2Θ ≤ 144.362°), 2963 unique (Rint = 0.0395, Rsigma = 0.0211) which were used in all calculations. The final R1 was 0.0264 (I > 2σ(I)) and wR2 was 0.0716 (all data). The goodness of fit on F^2 was 1.035. Flack parameter = 0.04(4). CCDC number 2130852, the data can be obtained free of charge from http://www.ccdc.cam.ac.uk/ (or from the Cambridge Crystallographic Data Centre, 12 Union Road, Cambridge CB2 1EZ, UK; fax: (+44) 1223-336-033; or deposit@ccdc.cam.ac.uk).

Inumakilactone B (2): ^{1}H NMR (500 MHz, DMSO-*d*$_6$): δ_H 6.51 (1H, s), 5.76 (1H, ddd, *J* = 17.5, 10.4, 7.2 Hz), 5.60 (1H, d, *J* = 17.2 Hz), 5.48 (1H, d, *J* = 10.4 Hz), 5.43 (1H, d, *J* = 7.3 Hz), 5.00 (1H, dd, *J* = 5.1, 1.2 Hz), 4.25 (1H, dd, *J* = 6.0, 5.2 Hz), 3.80 (1H, d, *J* = 1.2 Hz), 3.58 (1H, d, *J* = 4.3 Hz), 3.33 (1H, dd, *J* = 6.0, 4.1 Hz), 2.04 (1H, d, *J* = 5.2 Hz), 1.07 (3H, s), 1.33 (3H, s); ^{13}C NMR (125 MHz, DMSO-*d*$_6$): δ_C 175.7, 162.4, 156.7, 128.6, 123.2, 118.7, 79.8, 70.2, 67.0, 56.2, 54.8, 53.1, 50.2, 47.8, 44.4, 36.9, 24.6, 20.3.

Nagilactone C (3): ^{1}H NMR (500 MHz, DMSO-*d*$_6$): δ_H 6.27 (1H, s), 5.77 (1H, d, *J* = 4.5 Hz), 5.28 (1H, d, *J* = 4.9 Hz), 5.21 (1H, dd, *J* = 8.6, 4.4 Hz), 3.56 (1H, d, *J* = 4.4 Hz), 3.34 (1H, td, *J* = 6.0, 4.3 Hz), 3.29 (1H, m), 2.05 (1H, d, *J* = 6.8 Hz), 1.34 (3H, s), 1.31 (3H, s), 1.20 (3H, d, *J* = 6.8 Hz), 1.18 (3H, d, *J* = 6.8 Hz); ^{13}C NMR (150 MHz, pyridine-*d*$_5$): δ_C 177.8, 170.5, 165.3, 162.2, 111.9, 107.5, 73.9, 68.1, 60.3, 58.3, 51.7, 51.4, 50.3, 38.3, 29.8, 26.4, 20.9, 20.3, 19.4.

Nagilactone A (4): ^{1}H NMR (500 MHz, pyridine-*d*$_5$): δ_H 7.83 (1H, d, *J* = 3.5 Hz), 7.38 (1H, s), 6.76 (1H, d, *J* = 5.1 Hz), 5.69 (1H, dd, *J* = 8.5, 2.1 Hz), 5.20 (1H, dd, *J* = 8.6, 5.9 Hz), 4.18 (1H, dd, *J* = 12.4, 6.2 Hz), 3.52 (1H, m), 2.55 (1H, ddd, *J* = 14.0, 7.5, 6.0 Hz), 2.02–2.06 (2H, m), 2.03 (3H, s), 1.85 (1H, d, *J* = 5.9 Hz), 1.58 (1H, ddd, *J* = 14.5, 8.0, 6.5 Hz), 1.35 (3H, s), 1.34 (3H, d, *J* = 6.8 Hz), 1.28 (3H, d, *J* = 6.8 Hz); ^{13}C NMR (125 MHz, pyridine-*d*$_5$): δ_C 181.2, 169.5, 165.9, 162.7, 111.8, 107.9, 74.7, 70.8, 60.3, 50.0, 43.0, 41.4, 29.6, 29.4, 27.8, 24.4, 20.5, 20.0, 15.9.

Podolactone C (5): ^{1}H NMR (600 MHz, pyridine-d_5): δ_H 6.23 (1H, s), 5.28 (1H, brs), 5.07 (1H, m), 4.88 (1H, s), 3.79 (1H, d, *J* = 13.7 Hz), 3.44 (1H, d, *J* = 13.7 Hz), 3.41 (1H, brs), 3.30 (1H, d, *J* = 3.6 Hz), 2.69 (3H, s), 2.22 (1H, d, *J* = 14.7 Hz), 1.88 (3H, s), 1.83 (1H, d, *J* = 4.5 Hz), 1.77 (1H, d, *J* = 14.6 Hz), 1.45 (3H, s), 1.41 (3H, s). ^{13}C NMR (150 MHz, pyridine-d_5): δ_C 177.7, 163.6, 159.4, 150.7, 150.0, 136.4, 135.8, 124.4, 123.7, 117.4, 83.8, 80.3, 73.9, 73.7, 62.7, 59.0, 56.3, 53.4, 52.5, 50.2, 43.7, 43.4, 40.9, 36.3, 31.1, 28.3, 26.1, 21.6.

Nagilactone B (6): ^{1}H NMR (500 MHz, pyridine-d_5): δ_H 7.03 (1H, s), 5.65 (1H, d, *J* = 8.3 Hz), 5.17 (1H, m), 4.33 (1H, d, *J* = 7.1 Hz), 4.28 (1H, m), 3.49 (1H, m), 2.75 (1H, dd, *J* = 13.1, 13.1 Hz), 2.12 (1H, dd, *J* = 13.4, 4.5 Hz), 2.10 (3H, s), 1.91 (1H, d, *J* = 6.7 Hz), 1.47 (3H, s), 1.33 (3H, d, *J* = 6.9 Hz), 1.27 (3H, d, *J* = 6.8 Hz). ^{13}C NMR (125 MHz, pyridine-d_5): δ_C 182.2, 170.2, 167.0, 163.1, 123.7, 112.2, 108.2, 75.6, 71.9, 65.9, 60.5, 47.3, 43.3, 42.9, 35.6, 30.0, 24.4, 21.1, 20.7, 19.1.

15-Hydroxydehydroabietic acid (7): ^{1}H NMR (400 MHz, $CDCl_3$): δ_H 5.58 (1H, d, *J* = 3.3 Hz), 3.33 (1H, dd, *J* = 11.5, 4.3 Hz), 2.46 (1H, m), 2.05 (1H, m), 1.84 (1H, m), 1.66 (1H, m), 1.64 (1H, m), 1.50–1.62 (12H, m), 1.21 (3H, s), 1.20 (3H, s), 1.16 (3H, d, J = 7.6 Hz), 1.10 (3H, s); ^{13}C NMR (100 MHz, $CDCl_3$): δ_C 184.2, 148.0, 146.2, 134.9, 125.1, 124.3, 122.1, 72.5, 47.5, 44.7, 38.1, 37.1, 36.9, 31.8, 31.8, 30.3, 25.2, 21.9, 18.7, 16.4.

Dihydrovomifoliol (8): ^{1}H NMR (600 MHz, pyridine-d_5): δ_H 6.17 (1H, d, *J* = 4.3 Hz) , 6.13 (1H, s), 6.07 (1H, s), 3.99 (1H, brs), 2.77 (1H, d, *J* = 17.6 Hz), 2.40 (d, *J* = 17.6 Hz), 2.38 (1H, m), 2.22 (1H, ddd, *J* = 14.0, 11.7, 4.2 Hz), 2.17 (3H, s), 2.06 (1H, m), 1.82 (1H, dddd, *J* = 12.8, 12.8, 7.6, 5.3 Hz), 1.35 (3H, d, *J* = 6.1 Hz), 1.26 (3H, s), 1.21 (3H, s); ^{13}C NMR (150 MHz, pyridine-d_5): δ_C 198.0, 169.3, 126.6, 78.5, 68.4, 51.2, 42.7, 35.8, 35.7, 25.1, 24.8, 24.6, 22.1.

4*β*,10*α*-Aromadendranediol (9): ^{1}H NMR (600 MHz, $CDCl_3$): δ_H 1.80–1.89 (2H, m), 1.74 (1H, dd, *J* = 13.1, 7.0 Hz), 1.63–1.70 (3H, m), 1.60 (1H, m), 1.25 (3H, s), 1.20 (1H, t, *J* = 10.5 Hz), 1.18 (3H, s), 1.04 (6H, s), 0.88–0.94 (1H, m), 0.64 (1H, ddd, *J* = 11.3, 9.4, 6.2 Hz), 0.43 (1H, dd, *J* = 10.9, 9.4 Hz); ^{13}C NMR (150 MHz, $CDCl_3$): δ_C 80.5, 75.2, 56.6, 48.6, 44.6, 41.3, 28.8, 28.5, 26.8, 24.7, 24.0, 20.5, 20.4, 19.7, 16.6.

Eudesma-11(3)-ene-4*β*, 9*β*-diol (10): ^{1}H NMR (600 MHz, $CDCl_3$): δ_H 4.86 (1H, s), 4.60 (1H, s), 3.53 (1H, dd, *J* = 11.4, 4.8 Hz), 2.30 (1H, ddd, *J* = 13.8, 5.3, 1.9 Hz), 2.17 (1H, m), 2.11 (1H, m), 2.02 (1H, m), 1.84 (1H, dd, *J* = 14.0, 1.6 Hz), 1.80 (1H, m), 1.73 (1H, m), 1.38–1.55 (4H, m), 1.31 (1H, dd, *J* = 14.0, 10.3 Hz), 1.25 (3H, s), 1.24 (3H, s), 0.65 (3H, d, *J* = 0.6 Hz); ^{13}C NMR (150 MHz, $CDCl_3$): δ_C 146.0, 106.8, 79.5, 71.7, 58.0, 49.7, 47.7, 37.5, 34.8, 32.7, 32.0, 30.8, 30.3, 30.1,12.1.

Oplodiol (11): ^{1}H NMR (600 MHz, $CDCl_3$): δ_H 5.34 (1H, d, *J* = 4.6 Hz), 3.30 (1H, dd, *J* = 11.8, 3.9 Hz), 2.20 (1H, p, *J* = 6.8 Hz), 2.09 (1H, dd, *J* = 16.3, 5.3 Hz), 2.03 (1H, m), 1.83–1.88 (2H, m), 1.74 (1H, ddd, *J* = 14.1, 3.4, 3.4 Hz), 1.60 (1H, m), 1.54 (1H, ddd, *J* = 13.9, 13.9, 4.1 Hz), 1.30 (1H, dd, *J* = 11.9, 5.2 Hz), 1.18 (3H, s), 1.03 (3H, dd, *J* = 6.8 Hz), 1.02 (3H, dd, *J* = 6.8 Hz),

0.96 (3H, s); ^{13}C NMR (150 MHz, $CDCl_3$): δ_C 142.1, 116.2, 80.1, 71.1, 46.4, 40.9, 39.6, 37.8, 35.1, 30.0, 26.9, 23.2, 21.9, 21.4, 11.9.

Neriitide A (12): ^{1}H NMR (400 MHz, $CDCl_3$): δ_H 8.08 (1H, d, J = 9.0 Hz), 7.73 (1H, d, J = 15.5 Hz), 7.54 (2H, m), 7.43 (3H, m), 7.28 (2H, d, J = 7.5 Hz), 7.18 (1H, dd, J = 8.5, 2.0 Hz), 7.16 (1H, d, J = 8.0 Hz), 6.99–7.08 (5H, m), 6.73 (1H, m), 6.69 (1H, d, J = 15.5 Hz), 6.55 (1H, brd, J = 10.5 Hz), 6.35 (1H, d, J = 7.5 Hz), 6.05 (1H, d, J = 8.0 Hz), 4.94 (1H, dd, J = 6.6, 1.8 Hz), 4.61 (1H, ddd, J = 11.2, 7.9, 3.7 Hz), 4.32 (1H, dd, J = 9.2, 6.7 Hz), 4.15 (1H, d, J = 7.6 Hz), 3.58 (2H, m), 3.41 (1H, dd, J = 15.7, 3.5 Hz), 2.74 (dd, J = 15.7, 10.9 Hz), 2.26 (1H, dd, 12.2, 5.9 Hz), 2.03 (2H, m), 1.82 (1H, m), 1.60 (1H, m), 1.14 (3H, d, J = 6.8 Hz), 0.69 (3H, d, J = 6.6 Hz); ^{13}C NMR (100 MHz, $CDCl_3$): δ_C 171.6, 171.3, 167.0, 166.7, 156.1, 144.3, 136.8, 134.7, 132.0, 131.9, 130.5, 130.3, 129.1, 129.1, 128.7, 128.7, 128.4, 128.4, 128.2, 128.2, 126.7, 125.6, 123.7, 123.6, 117.1, 115.6, 82.0, 59.2, 55.3, 53.5, 47.3, 36.1, 29.2, 26.0, 25.1, 20.6, 14.7.

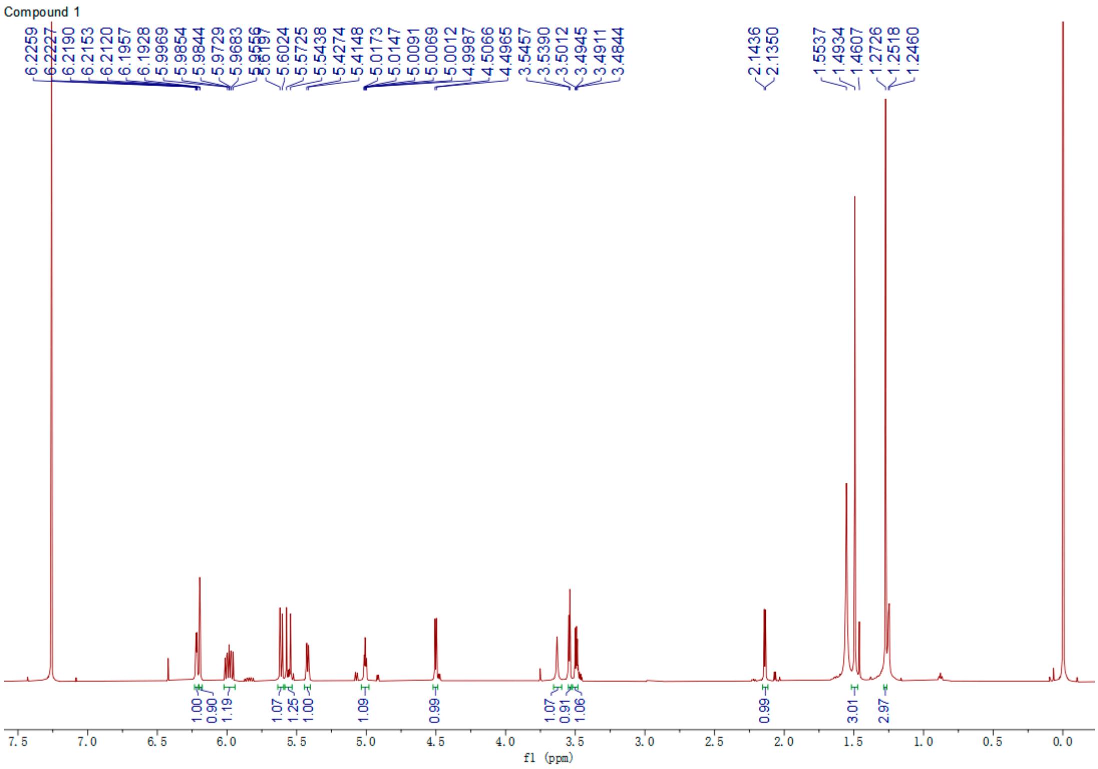

Figure S1. ^{1}H NMR spectrum of **1.**

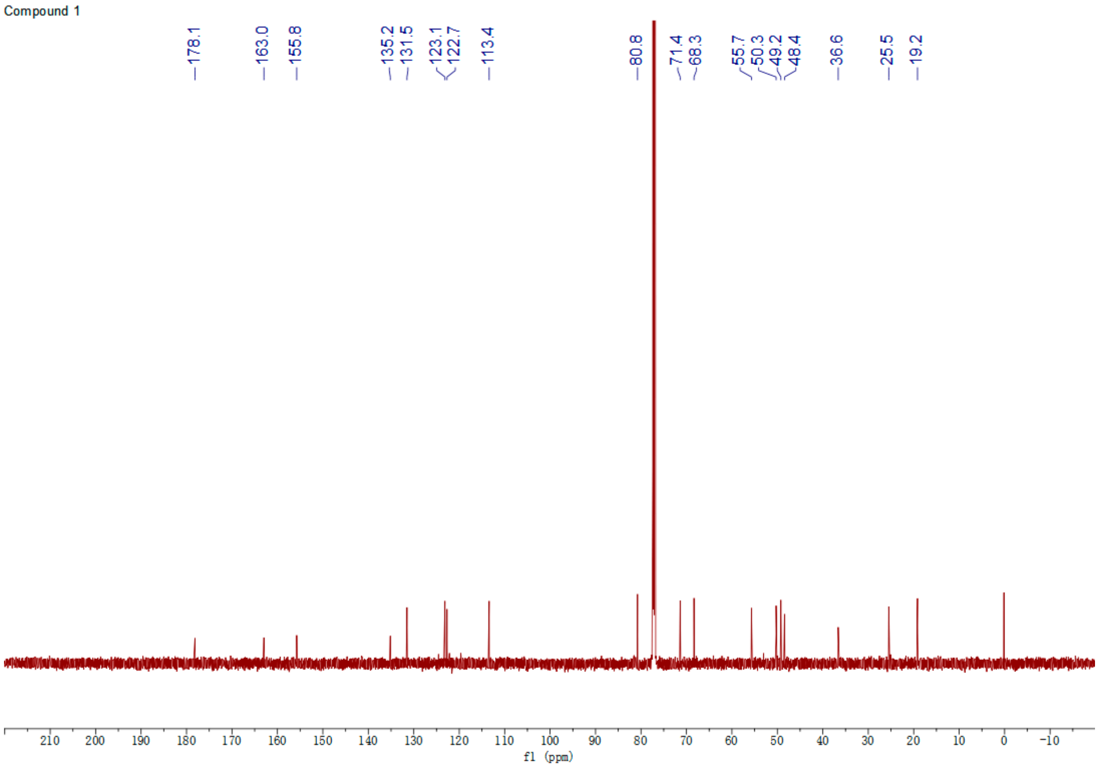

Figure S2. ^{13}C NMR spectrum of **1.**

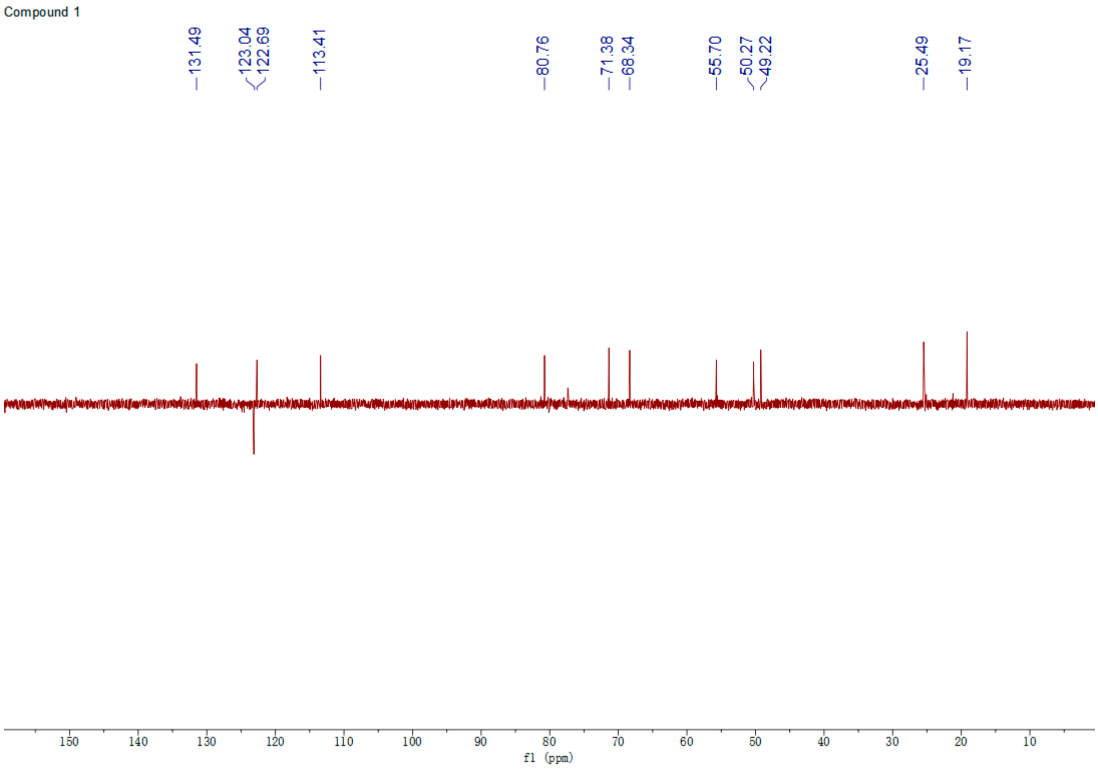

Figure S3. DEPT spectrum of **1.**

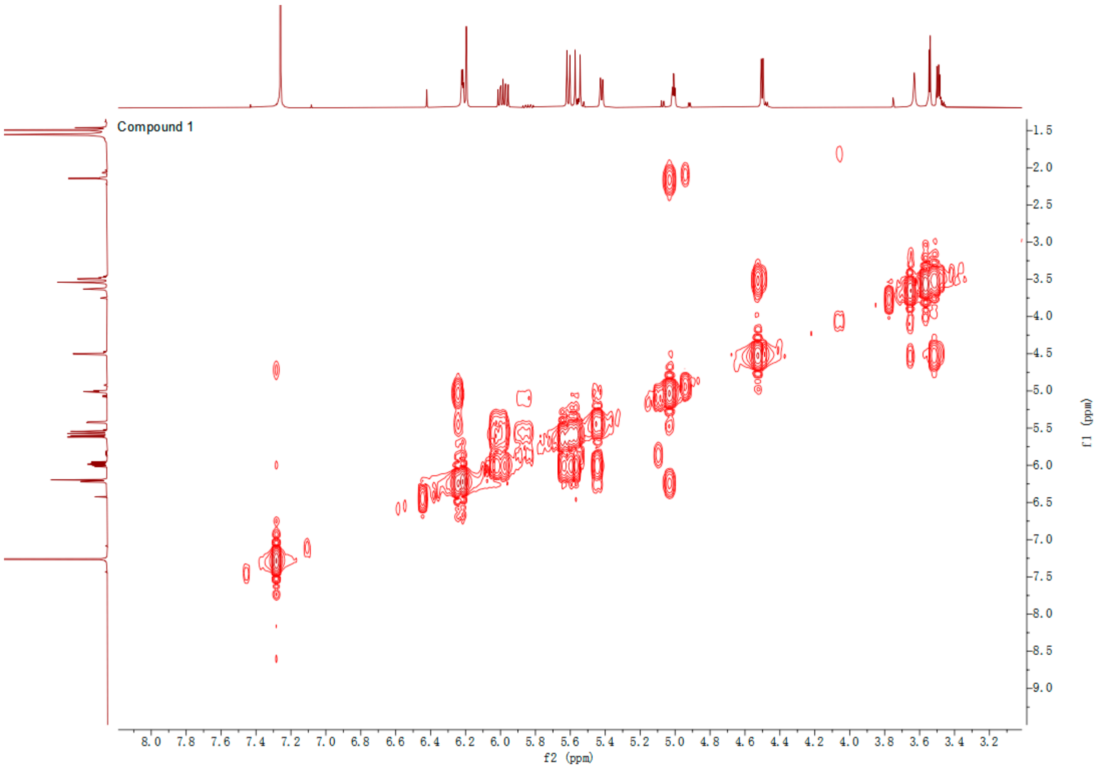

Figure S4. ^{1}H-^{1}H COSY spectrum of **1.**

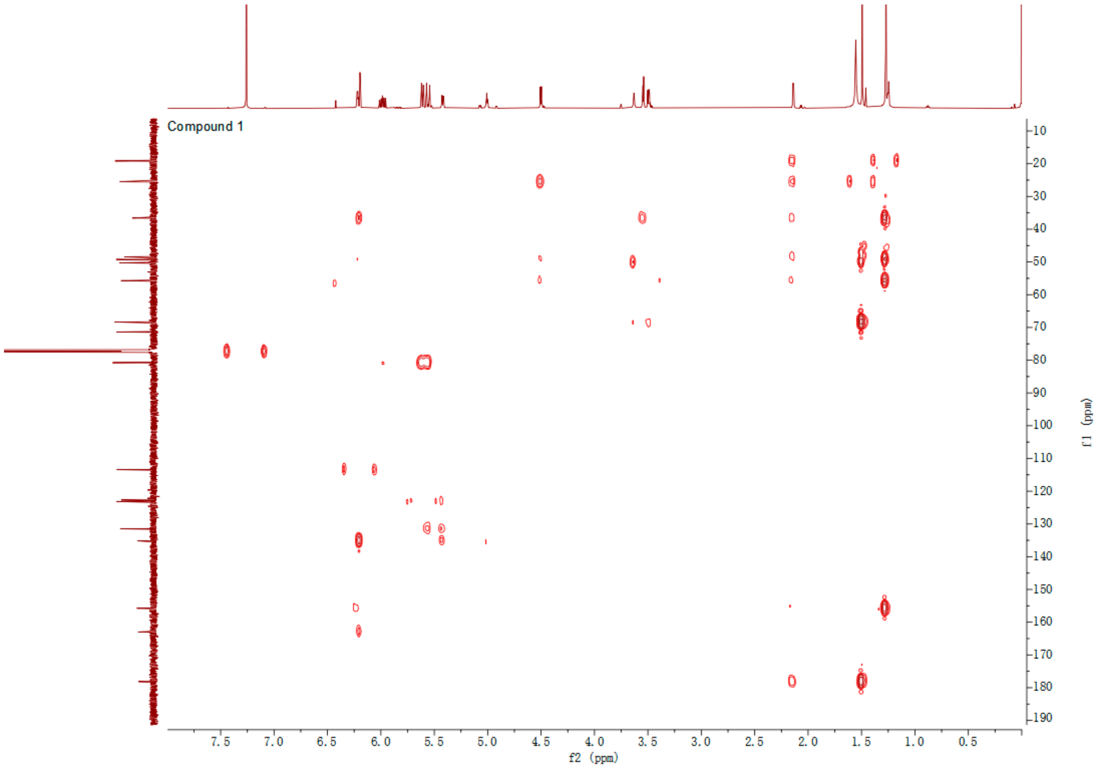

Figure S5. HMBC spectrum of **1.**

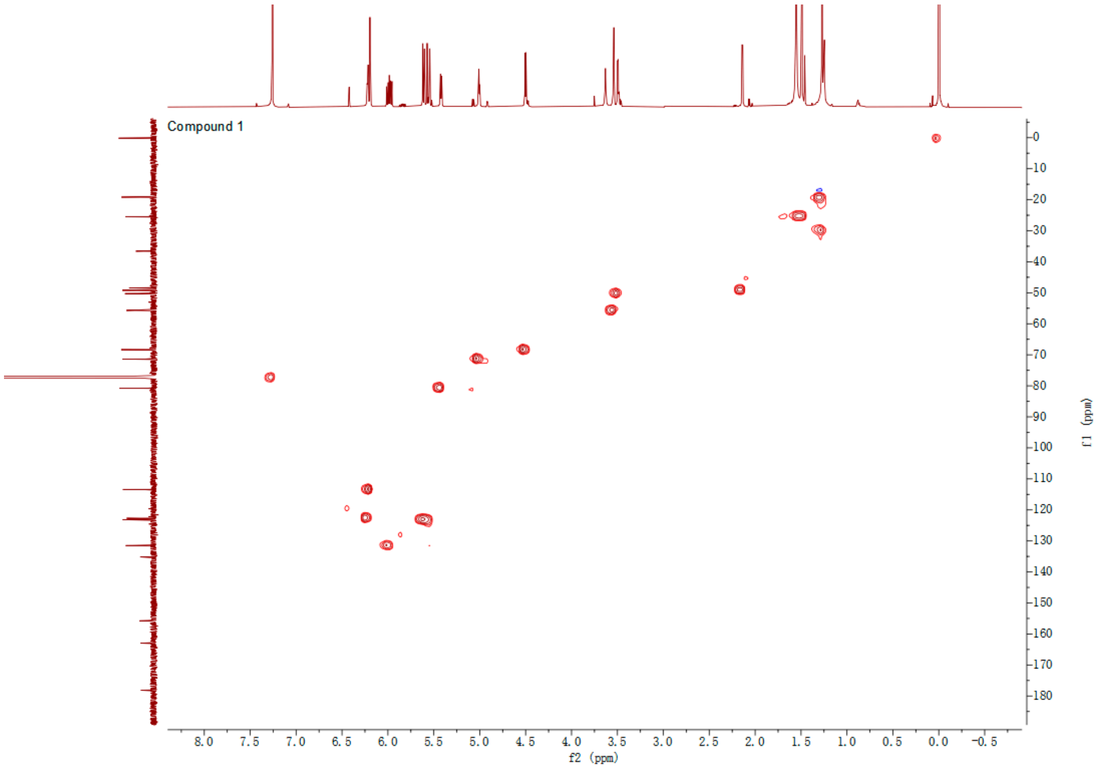

Figure S6. HSQC spectrum of **1.**

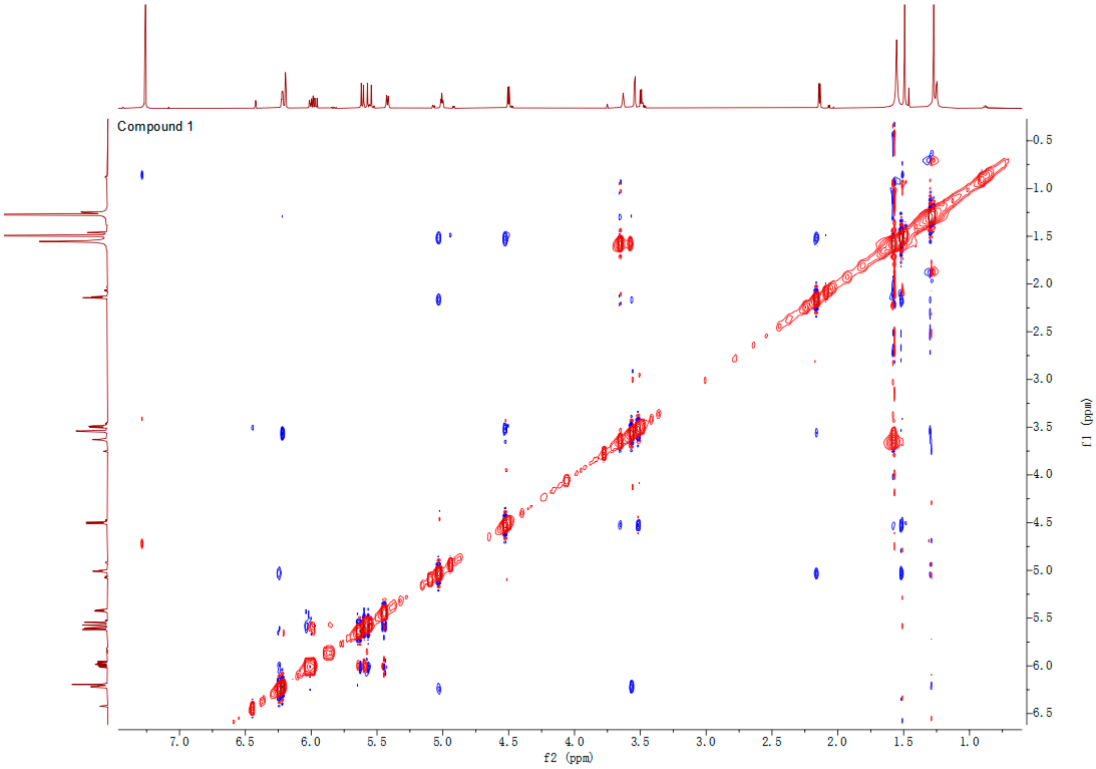

Figure S7. NOESY spectrum of **1.**

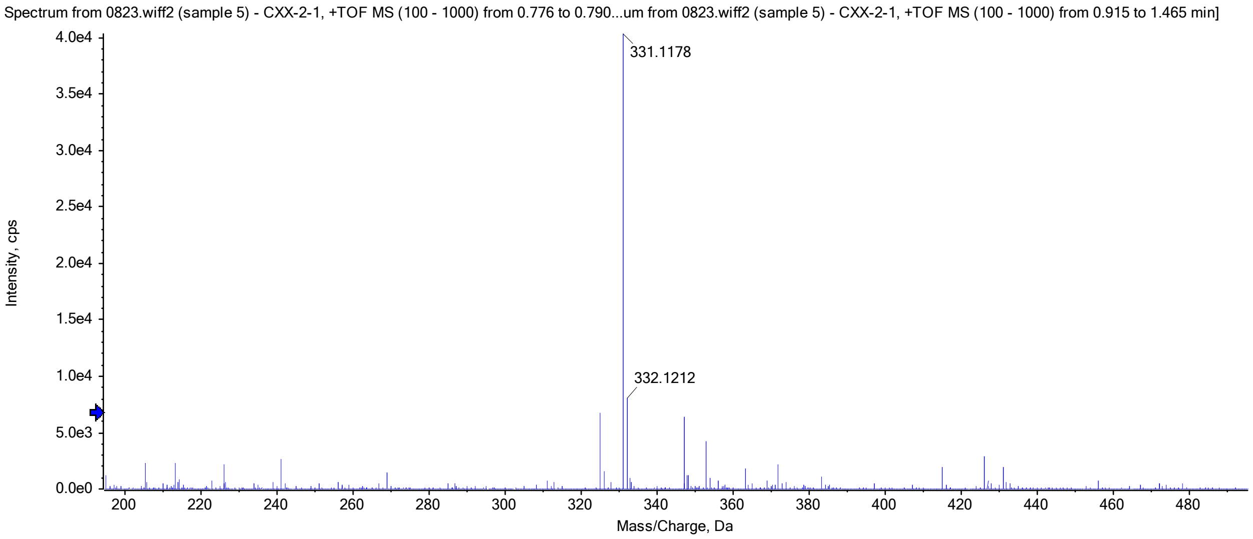

Figure S8. HRESIMS of **1.**

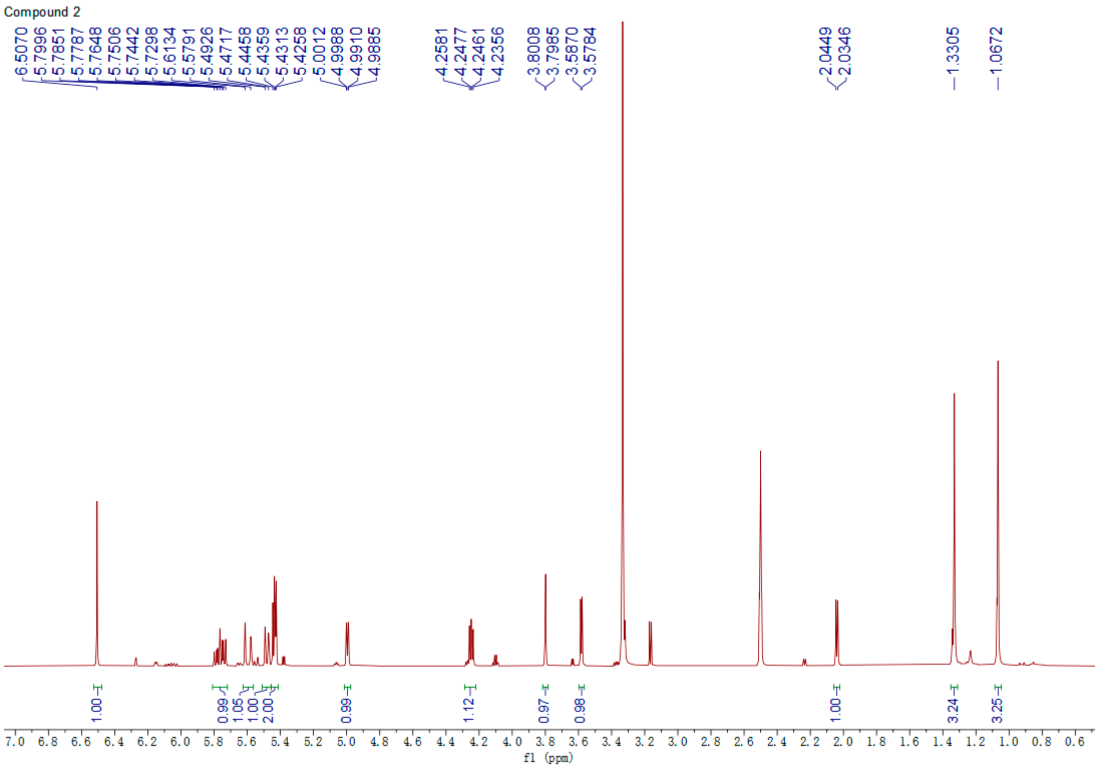

Figure S9. ^{1}H NMR spectrum of **2.**

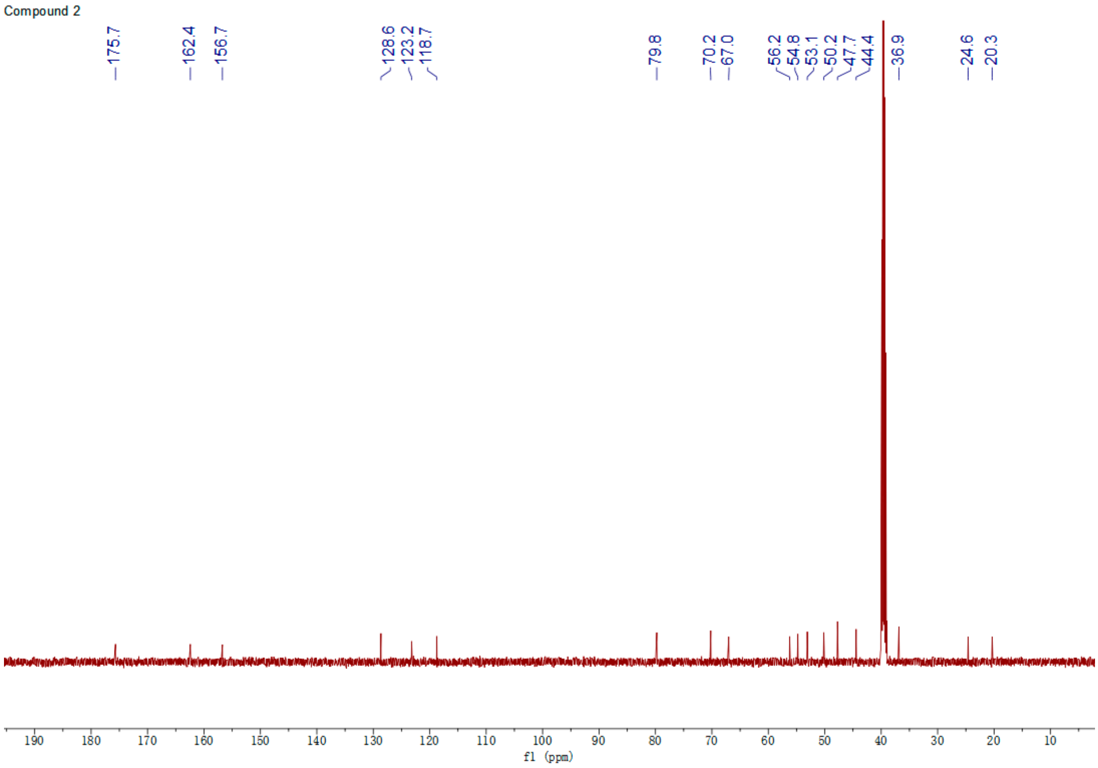

Figure S10. ^{13}C NMR spectrum of **2.**

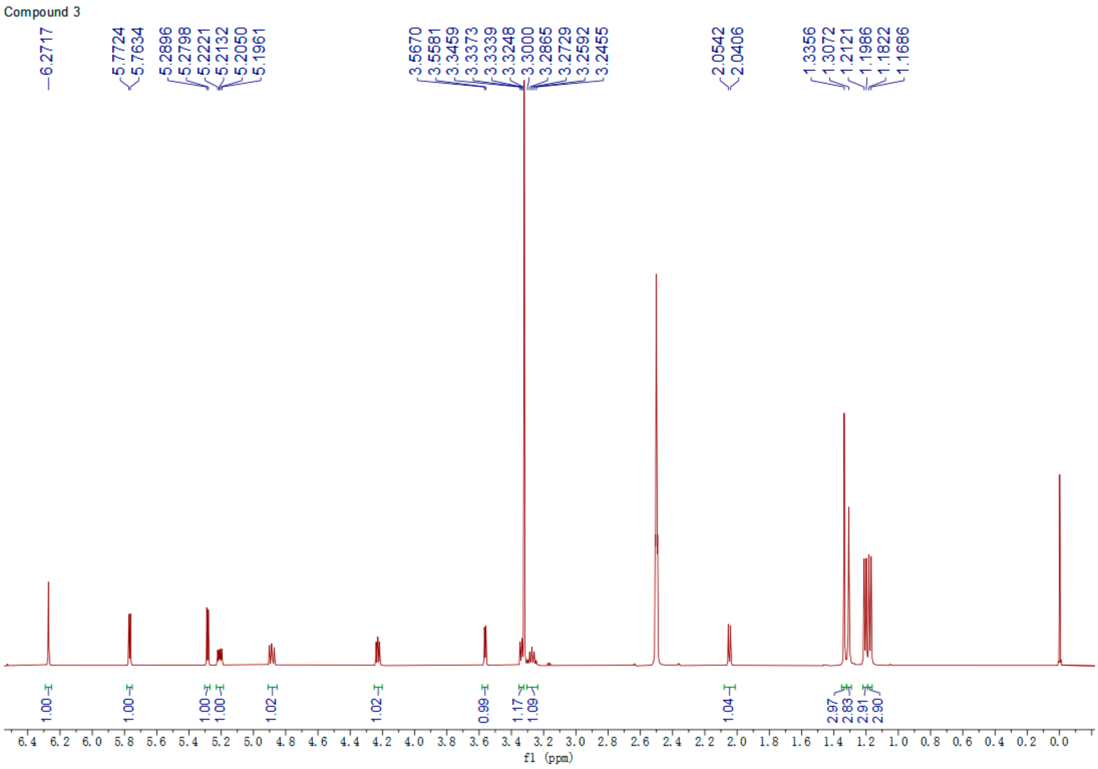

Figure S11. ^{1}H NMR spectrum of **3.**

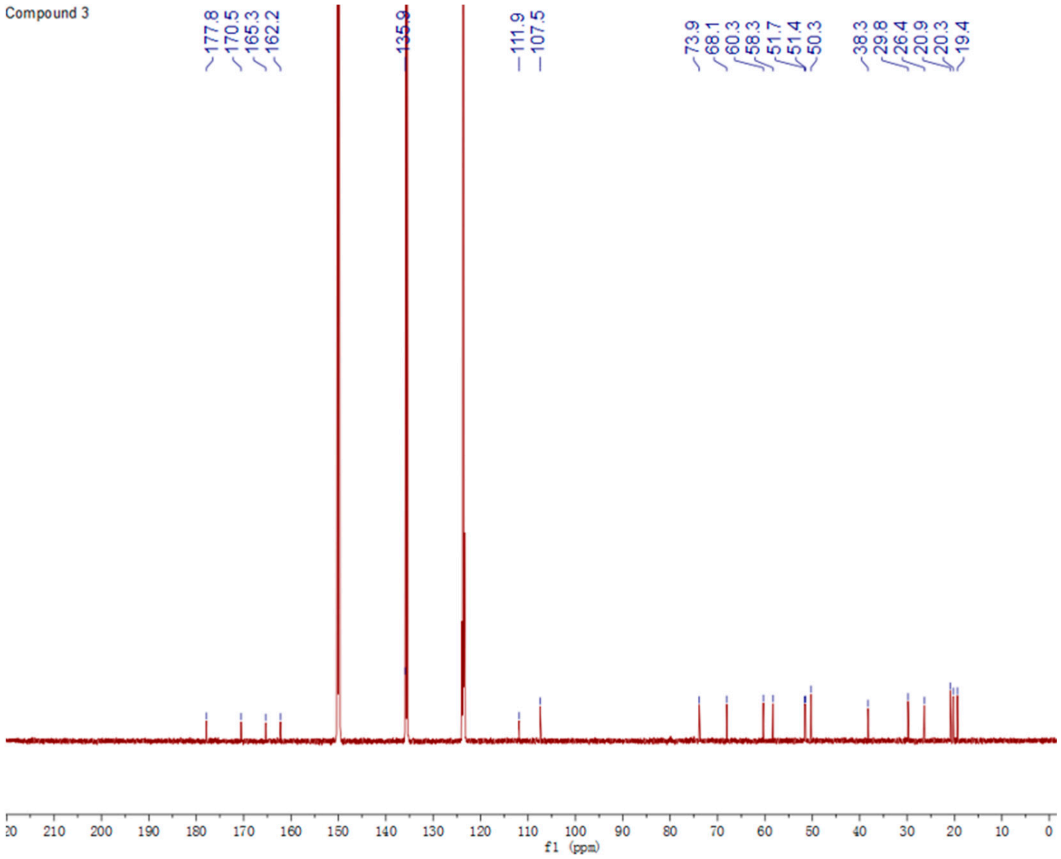

Figure S12. ^{13}C NMR spectrum of **3.**

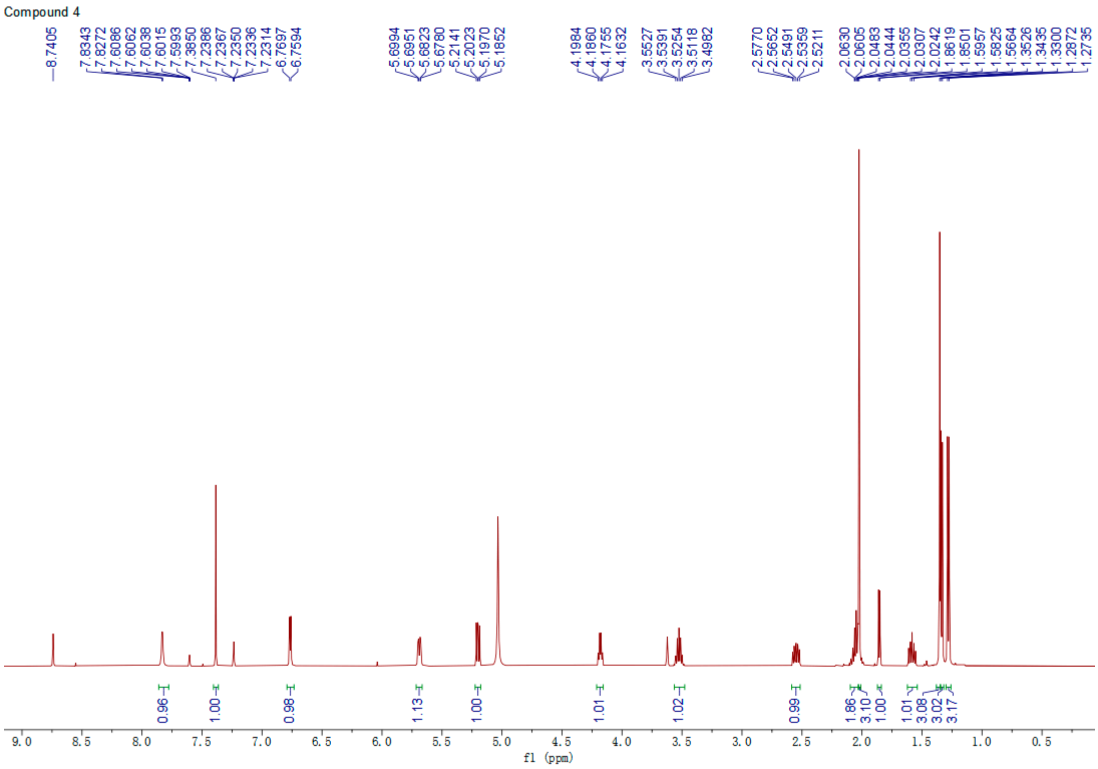

Figure S13. ^{1}H NMR spectrum of **4.**

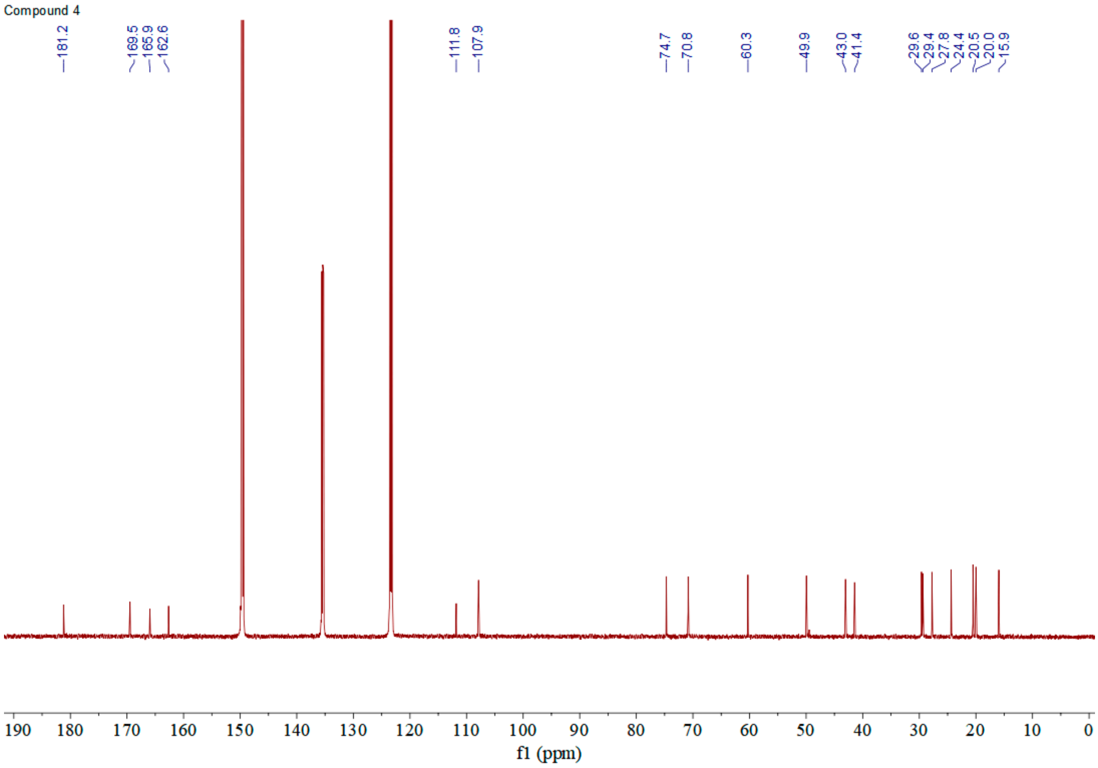

Figure S14. ^{13}C NMR spectrum of **4.**

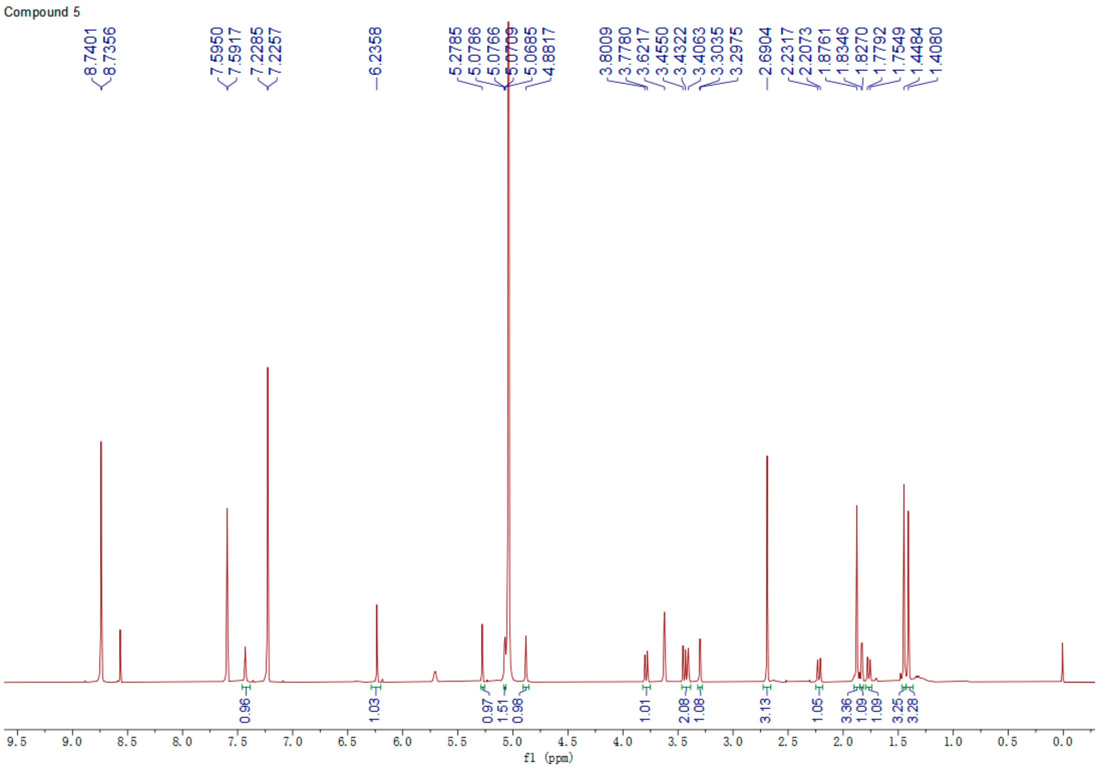

Figure S15. ^{1}H NMR spectrum of **5.**

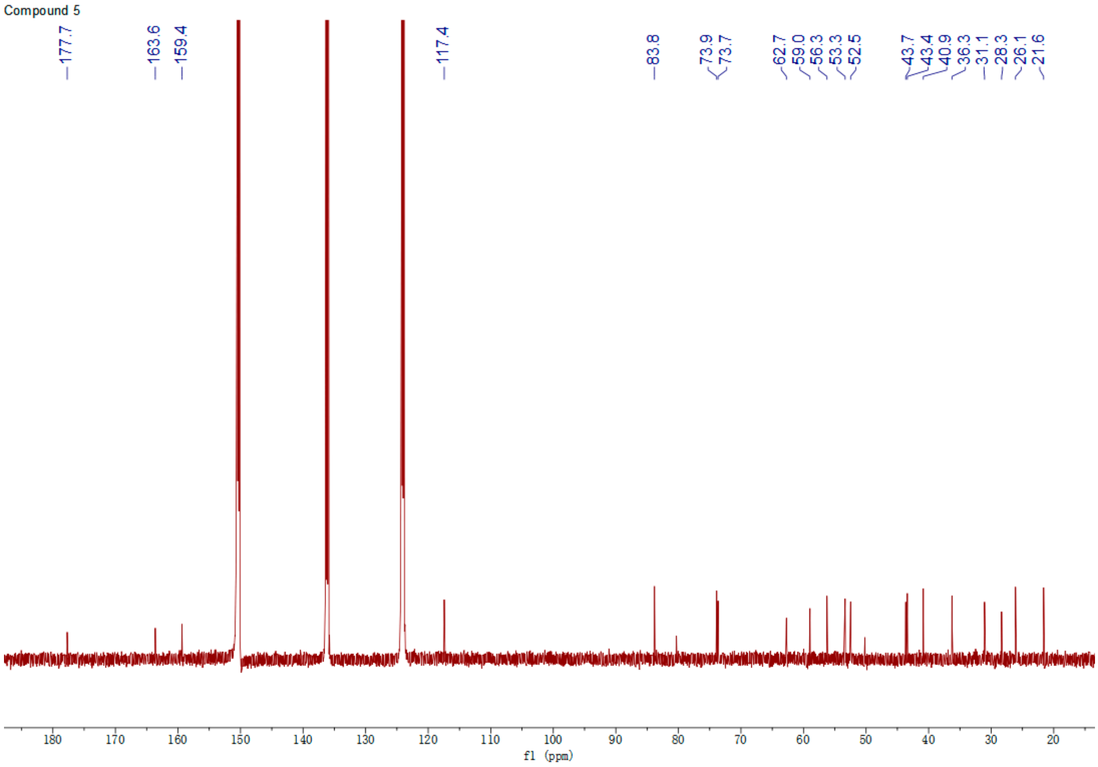

Figure S16. ^{13}C NMR spectrum of **5.**

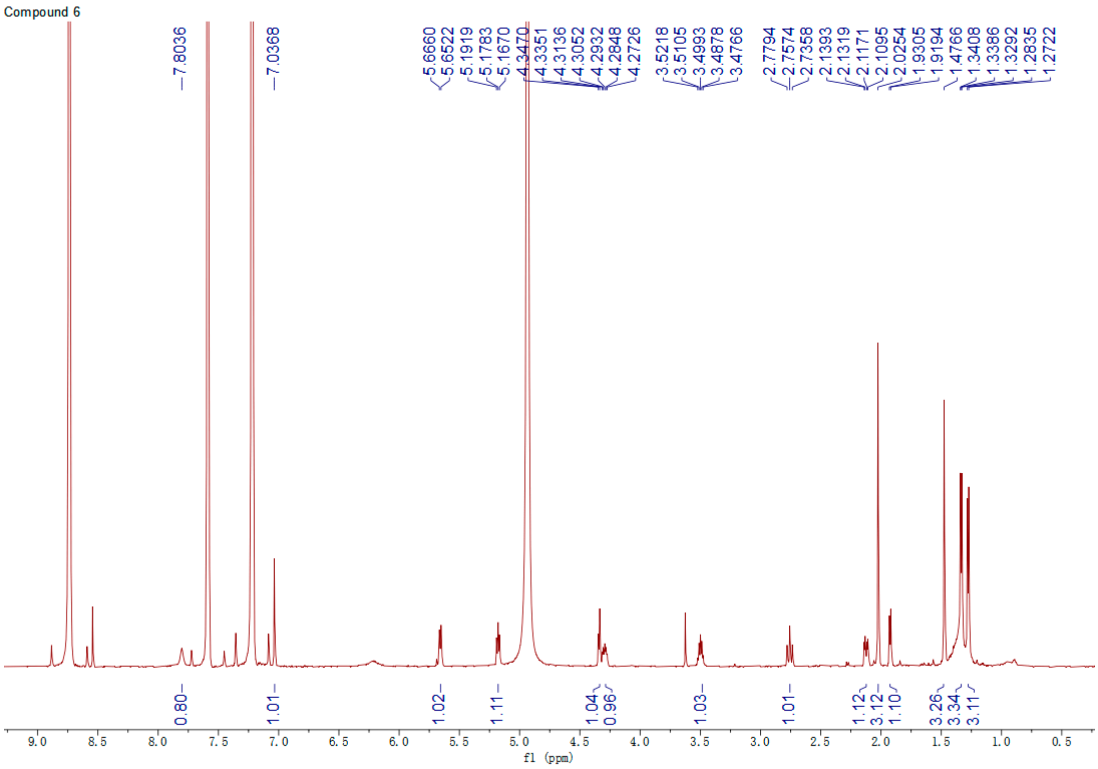

Figure S17. ^{1}H NMR spectrum of **6.**

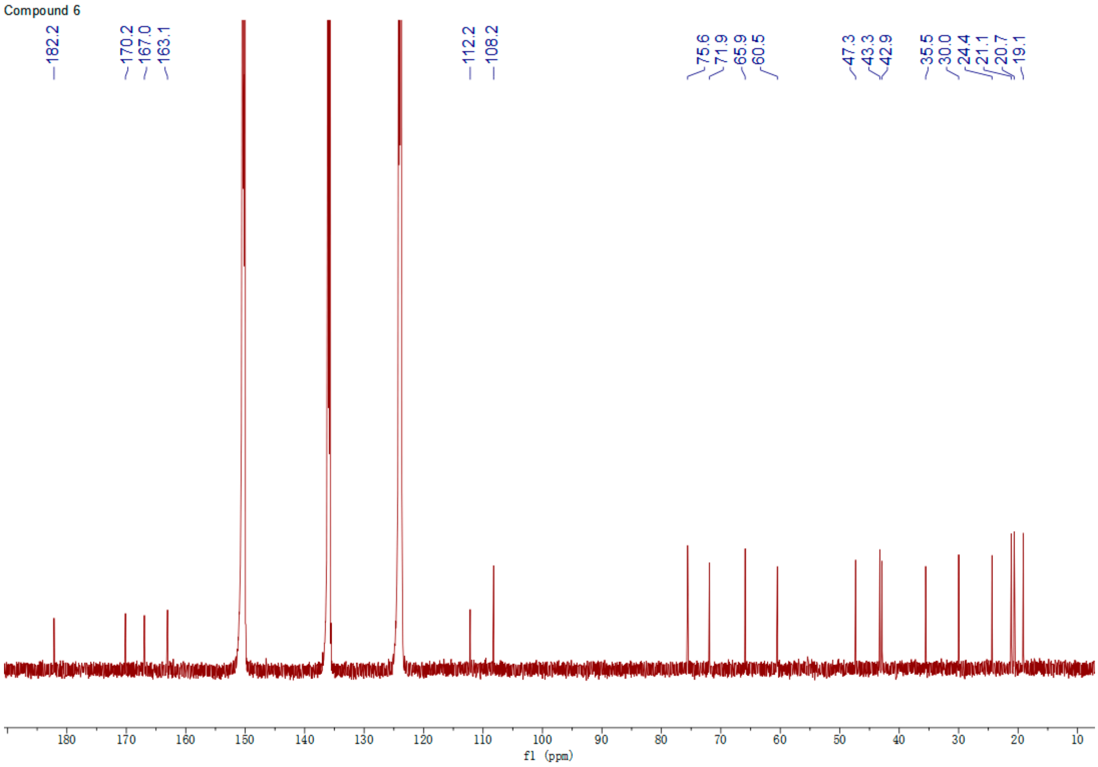

Figure S18. ^{13}C NMR spectrum of **6.**

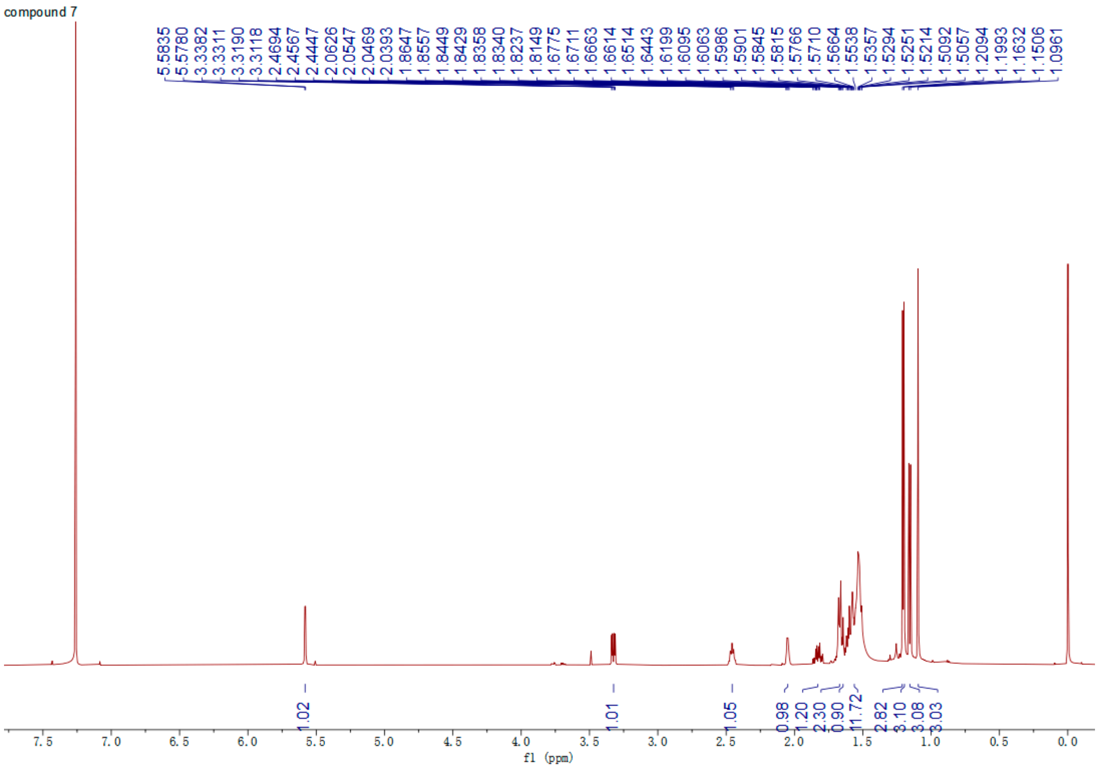

Figure S19. ^{1}H NMR spectrum of **7.**

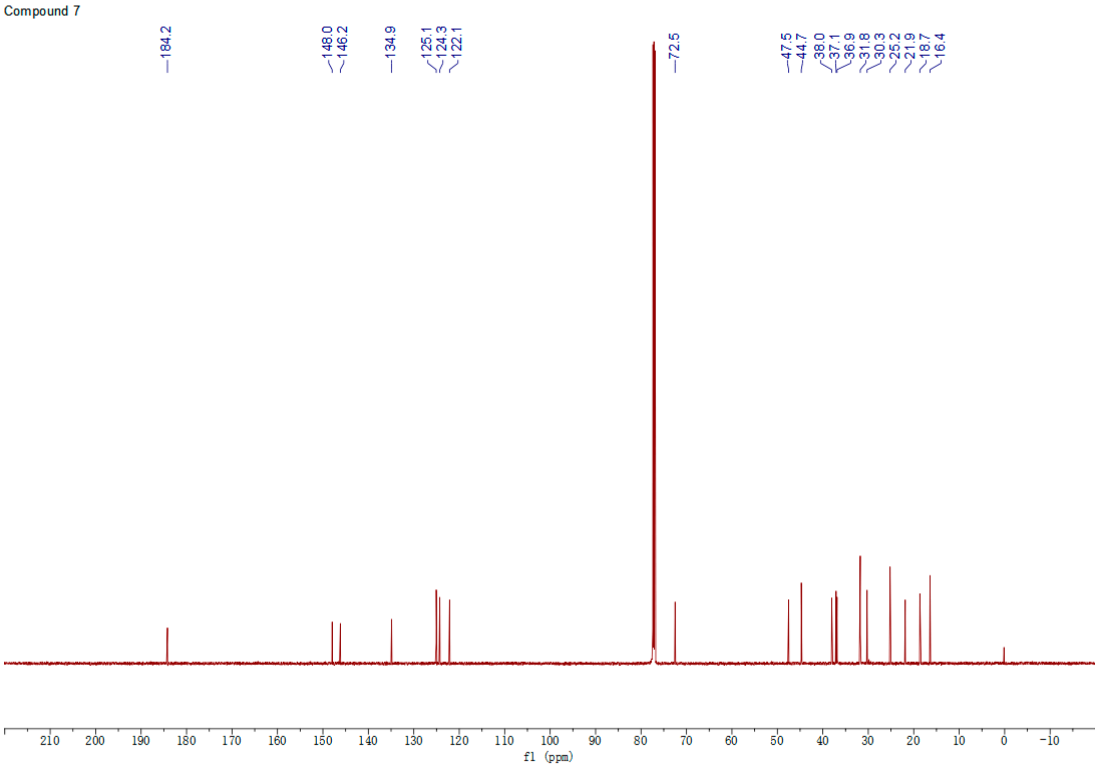

Figure S20. ^{13}C NMR spectrum of **7.**

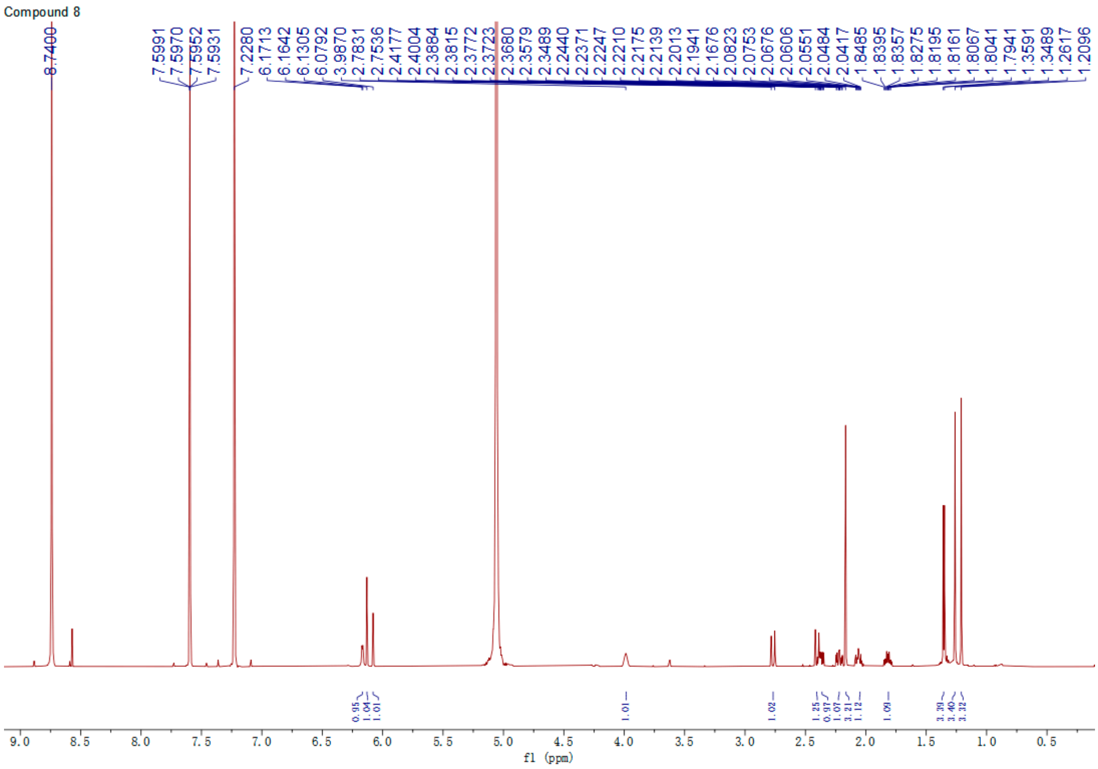

Figure S21. ^{1}H NMR spectrum of **8.**

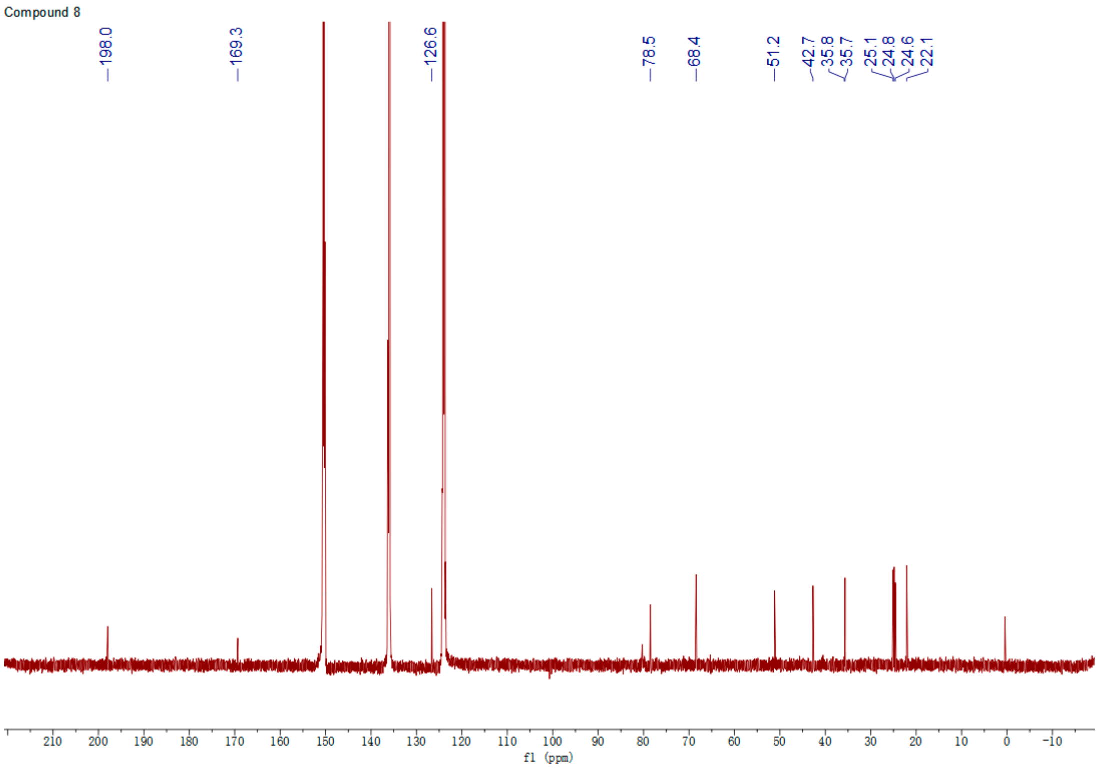

Figure S22. ^{13}C NMR spectrum of **8.**

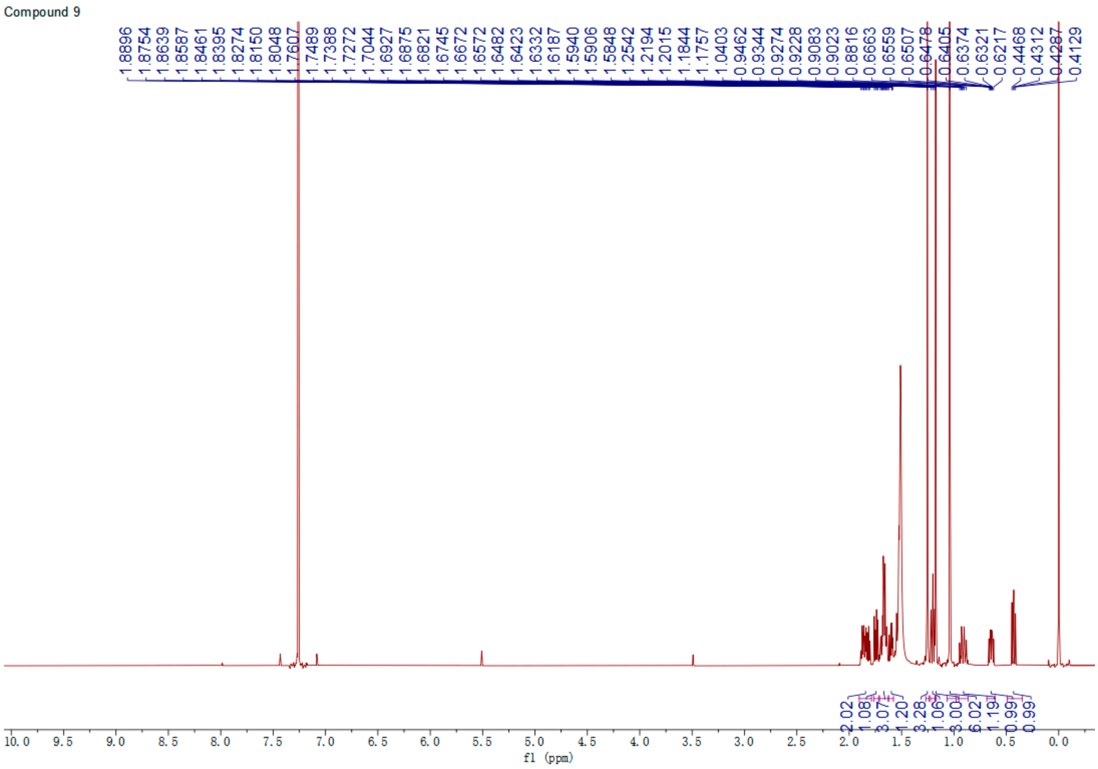

Figure S23. ^{1}H NMR spectrum of **9.**

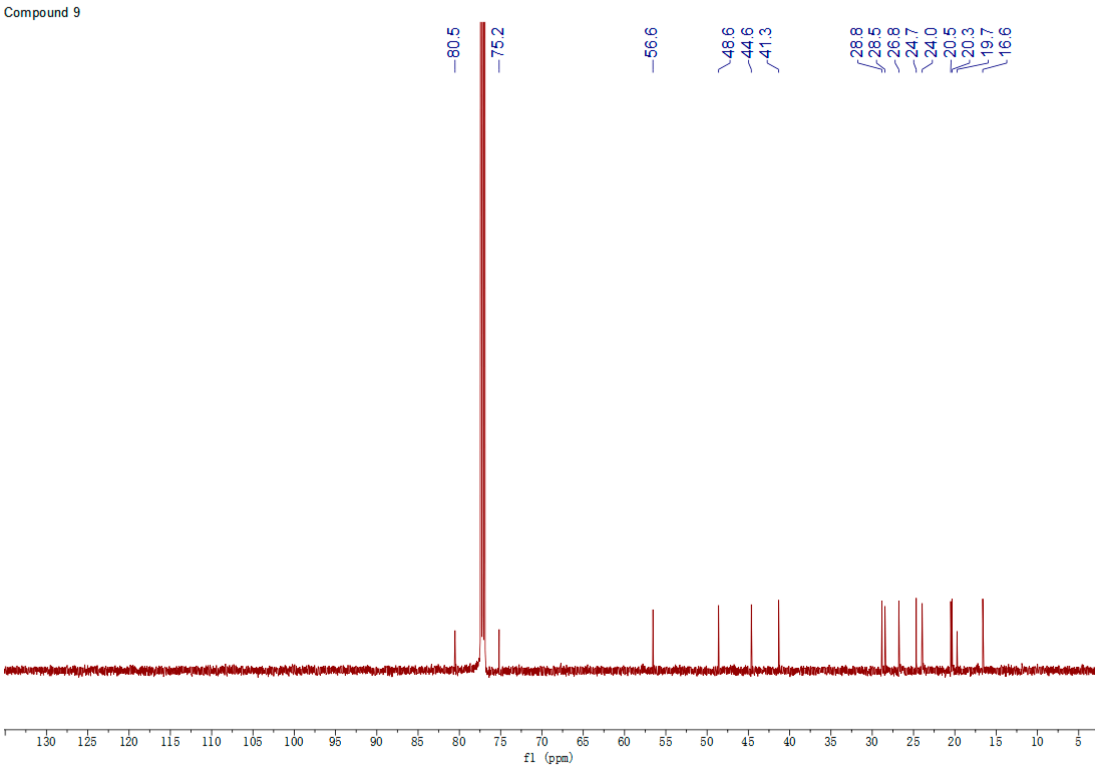

Figure S24. ^{13}C NMR spectrum of **9.**

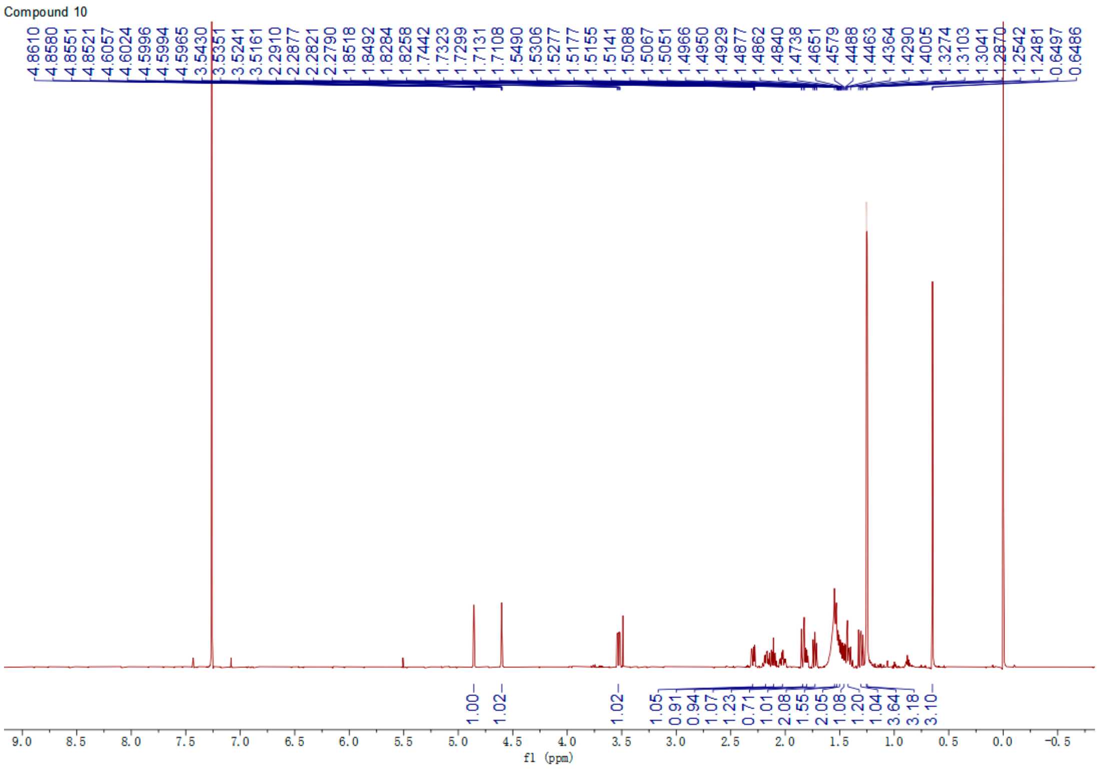

Figure S25. ^{1}H NMR spectrum of **10.**

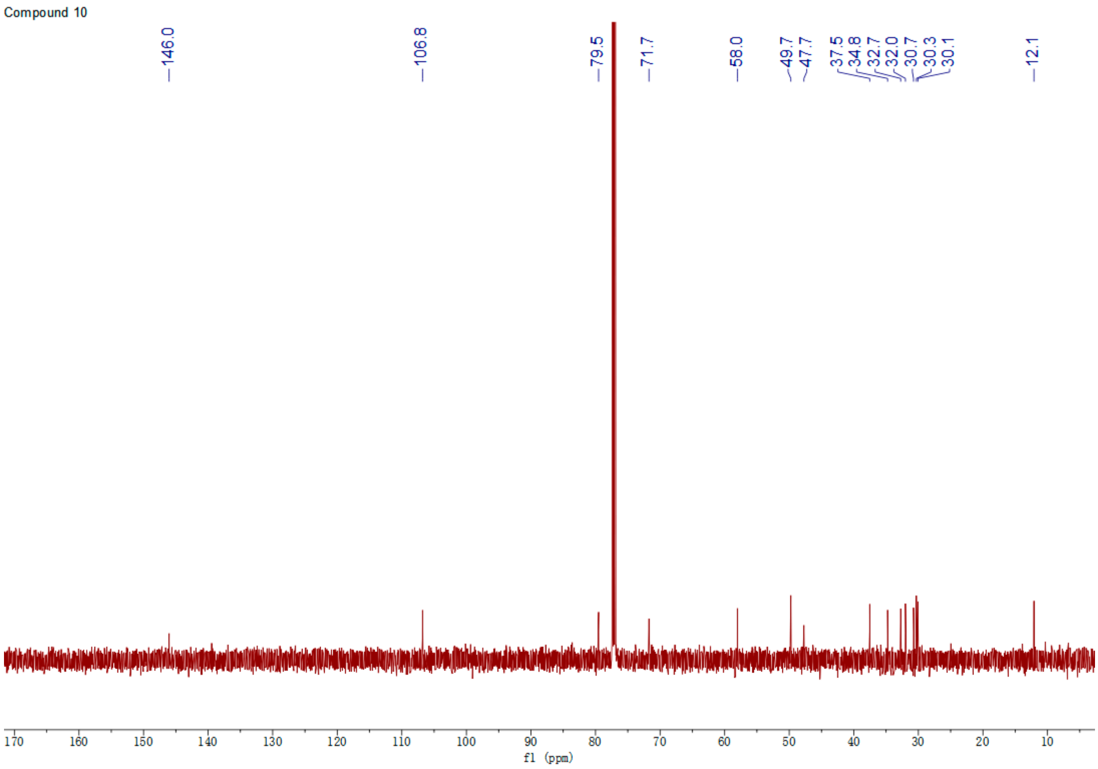

Figure S26. ^{13}C NMR spectrum of **10.**

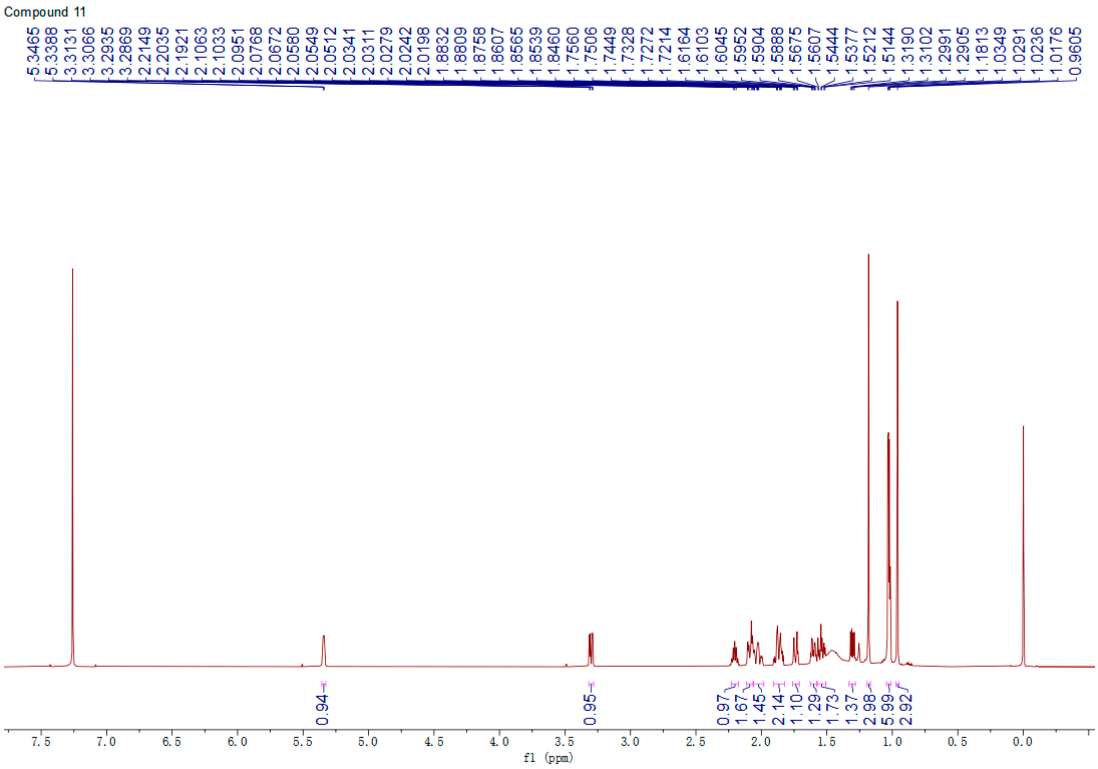

Figure S27. ^{1}H NMR spectrum of **11.**

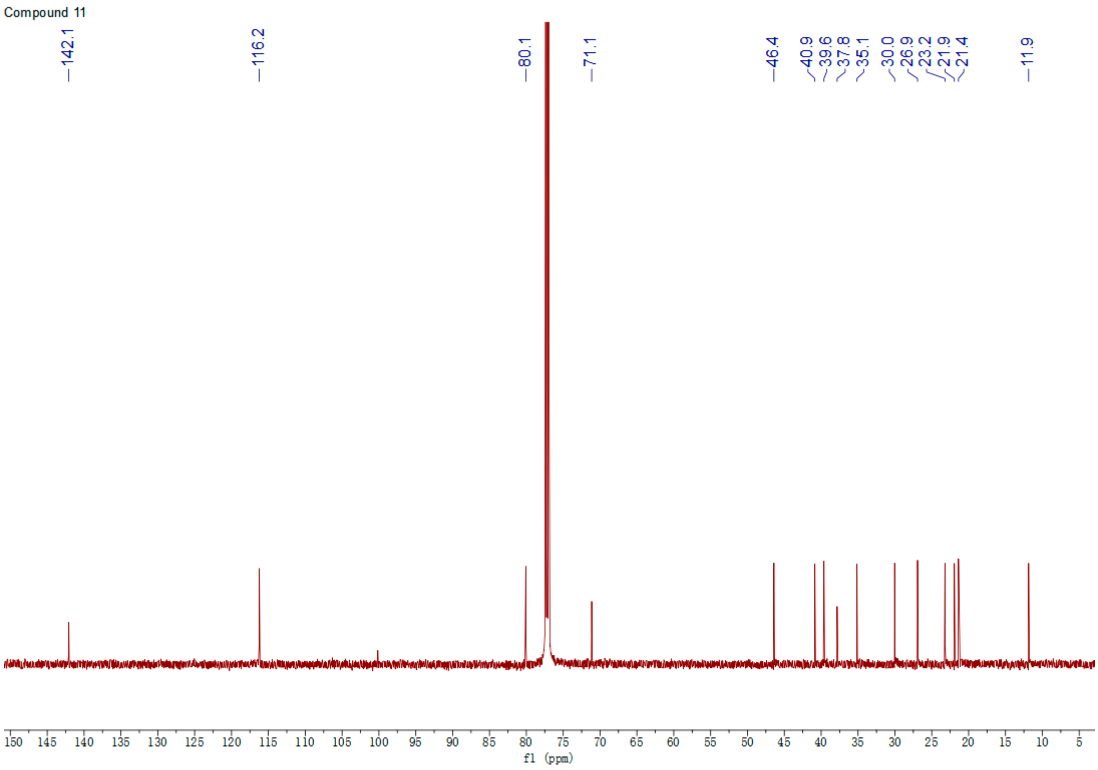

Figure S28. ^{13}C NMR spectrum of **11.**

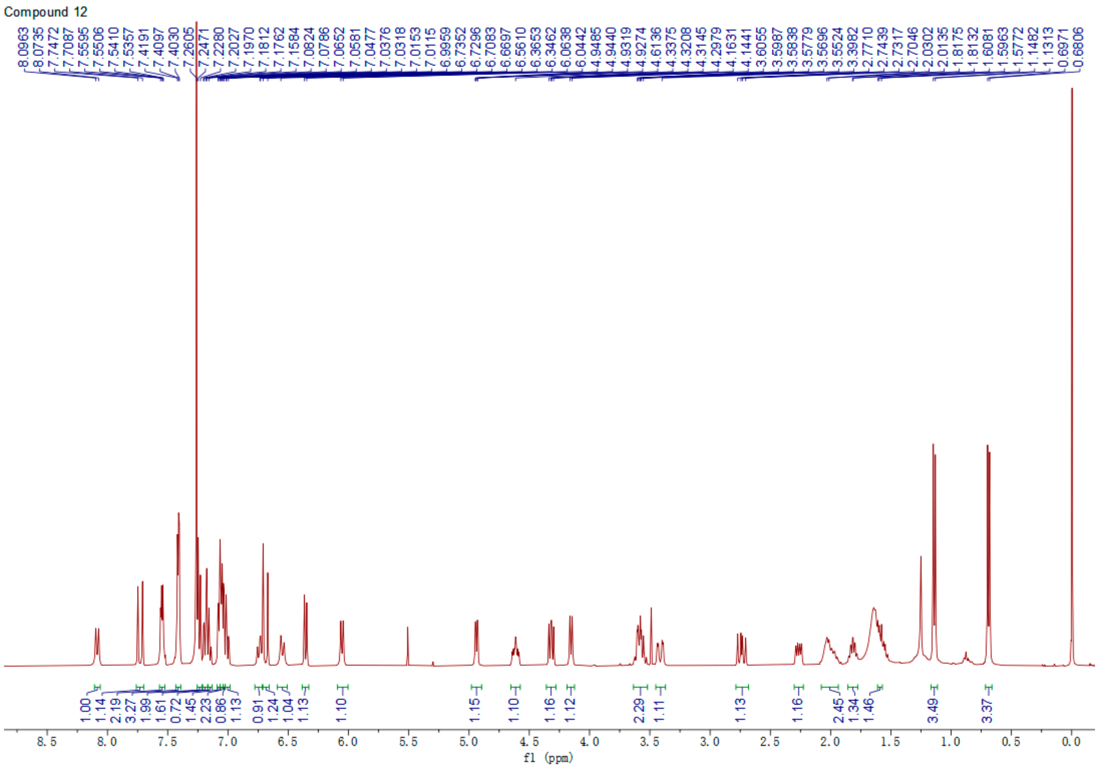

Figure S29. ^{1}H NMR spectrum of **12.**

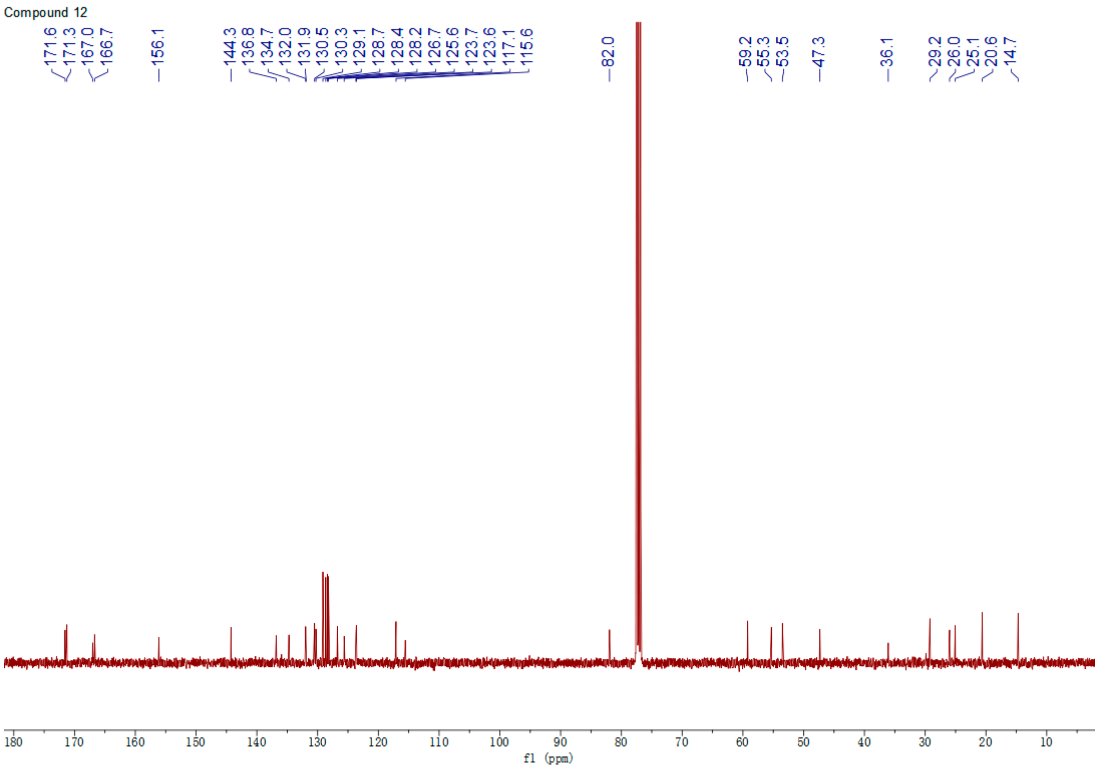

Figure S30. ^{13}C NMR spectrum of **12.**

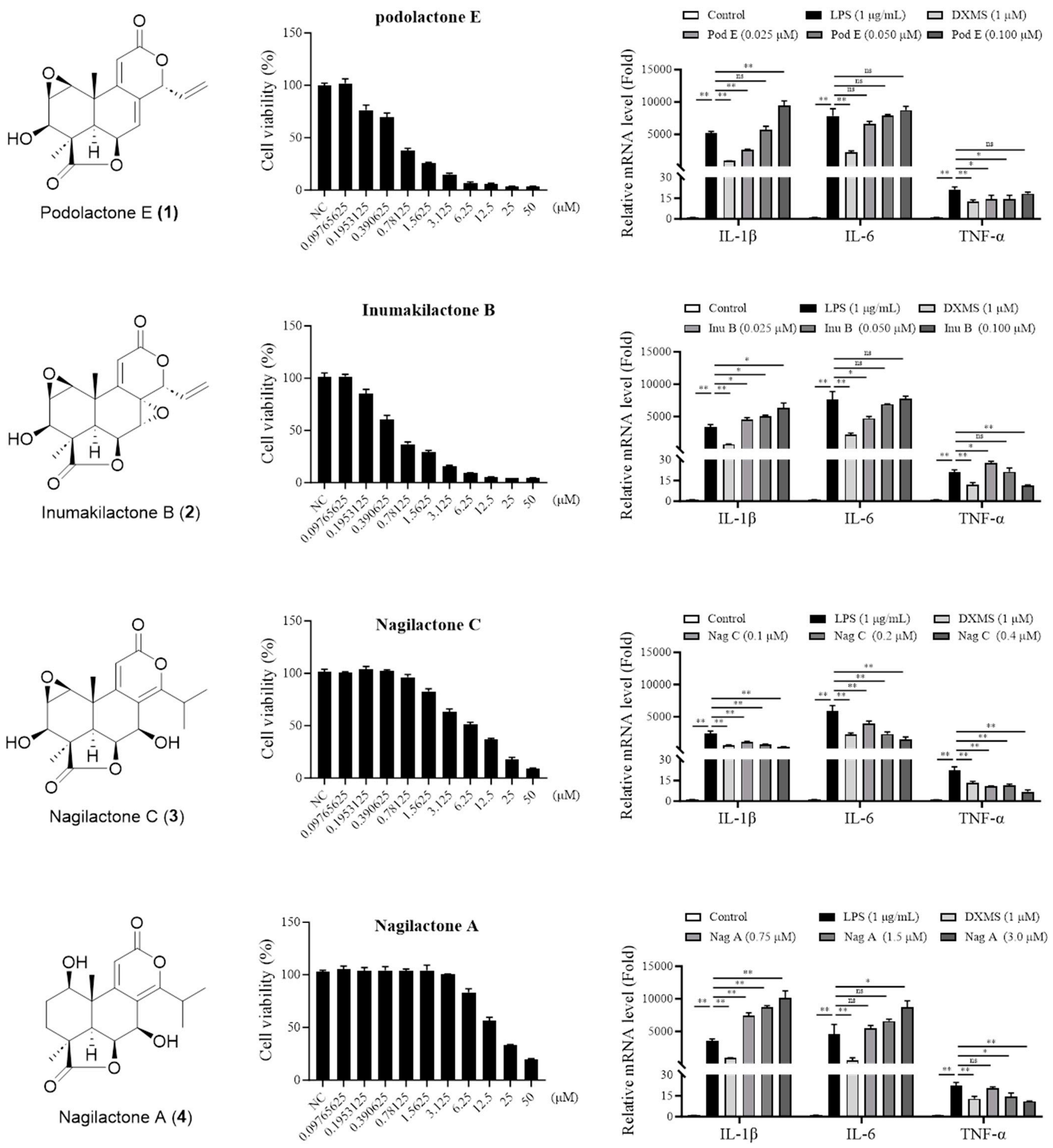

Figure S31. Cytotoxicity testing and anti-inflammatory activity screening of **1–4**.
(*, $p < 0.05$; **, $p < 0.01$; ns, $p > 0.05$, $n = 3$)

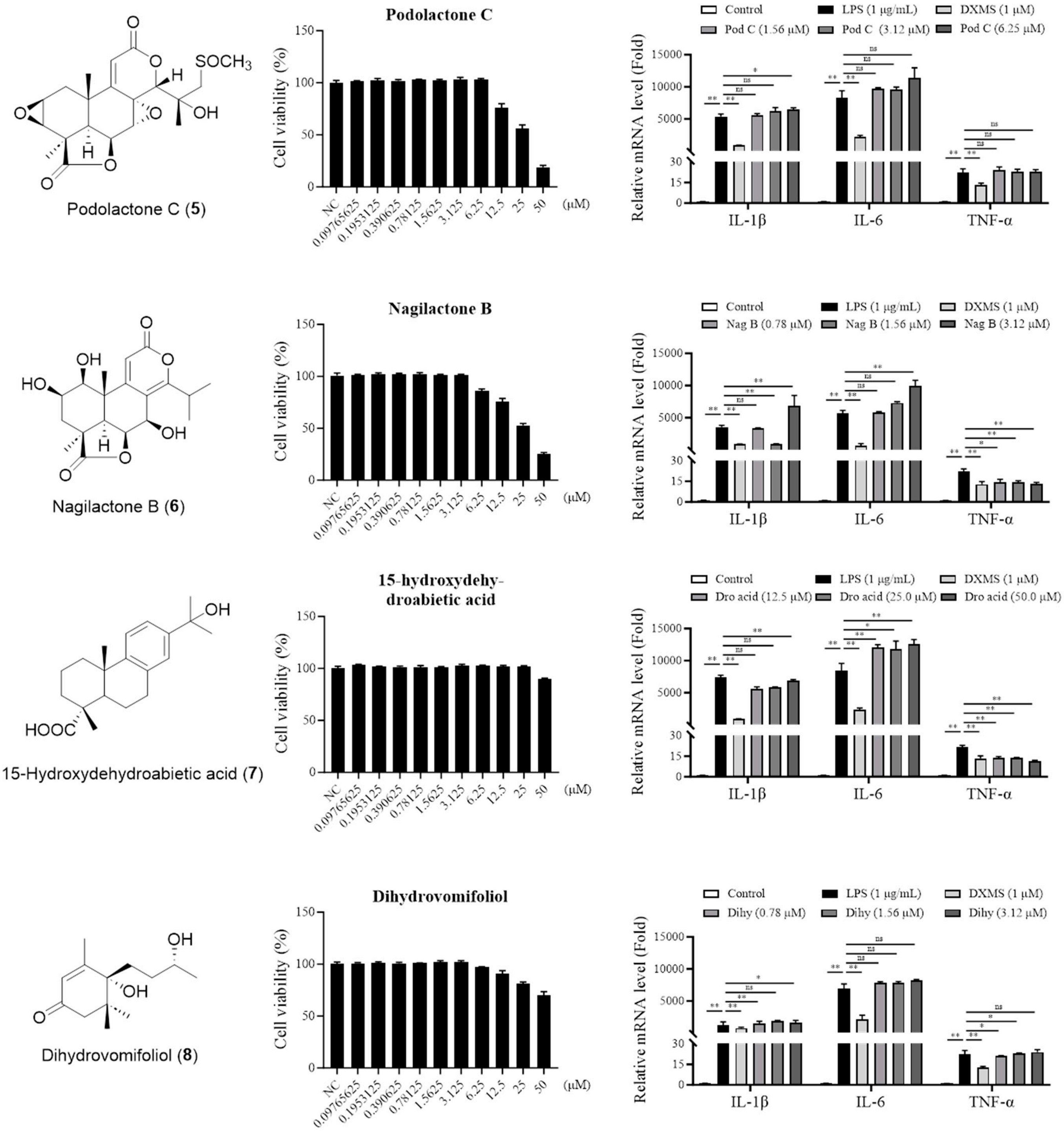

Figure S32. Cytotoxicity testing and anti-inflammatory activity screening of **5–8**.

(*, $p < 0.05$; **, $p < 0.01$; ns, $p > 0.05$, $n = 3$)

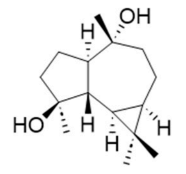

4β,10α-Aromadendranediol (**9**)

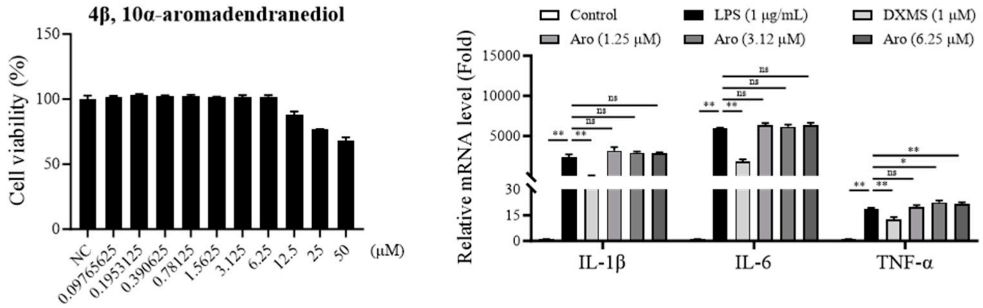

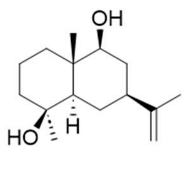

Eudesma-11(3)-ene-4β,9β-diol (**10**)

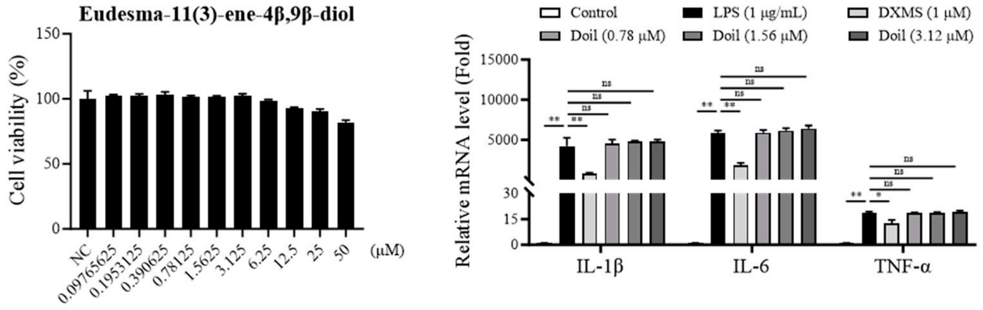

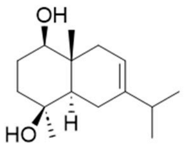

Oplodiol (**11**)

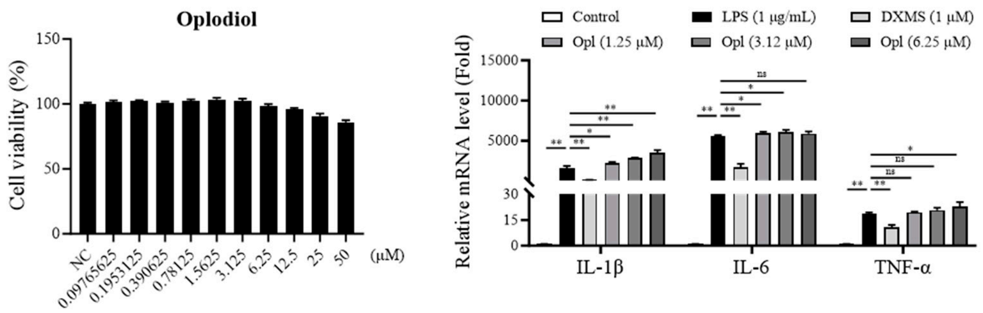

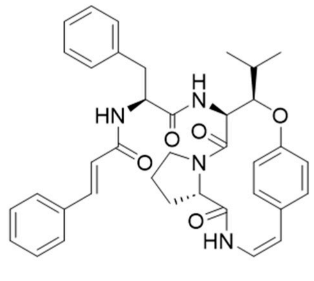

Neriitide A (**12**)

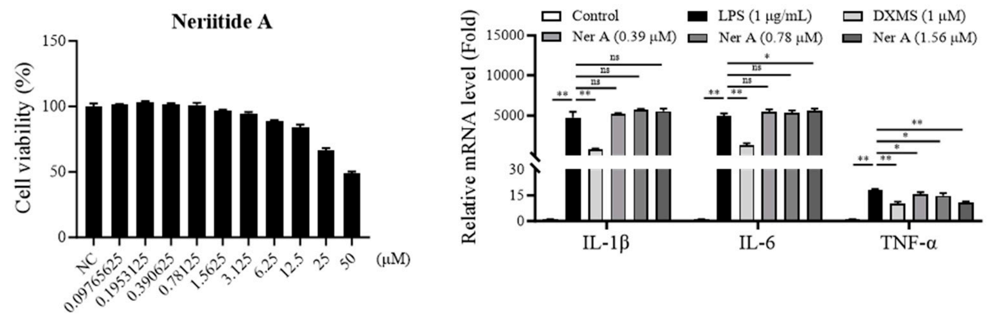

Figure S33. Cytotoxicity testing and anti-inflammatory activity screening of **9–12**.

(*, $p < 0.05$; **, $p < 0.01$; ns, $p > 0.05$, $n = 3$)
